# Protective role of host complement system in *Aspergillus fumigatus* infection

**DOI:** 10.3389/fimmu.2022.978152

**Published:** 2022-09-23

**Authors:** Rajashri Shende, Sarah Sze Wah Wong, Heikrujam Thoihen Meitei, Girdhari Lal, Taruna Madan, Vishukumar Aimanianda, Jayanta Kumar Pal, Arvind Sahu

**Affiliations:** ^1^ Complement Biology Laboratory, National Centre for Cell Science, Savitribai Phule (S. P.) Pune University Campus, Pune, India; ^2^ Dr. D.Y. Patil Biotechnology & Bioinformatics Institute, Dr. D.Y. Patil Vidyapeeth, Pune, India; ^3^ Institut Pasteur, Université Paris Cité, CNRS UMR2000, Unité Mycologie Moléculaire, Department of Mycology, Paris, France; ^4^ Laboratory of Autoimmunity and Tolerance, National Centre for Cell Science, Savitribai Phule (S. P.) Pune University Campus, Pune, India; ^5^ Department of Innate Immunity, ICMR – National Institute for Research in Reproductive and Child Health, Mumbai, India

**Keywords:** *Aspergillus fumigatus*, complement system, infectious disease, innate immunity, systemic aspergillosis

## Abstract

Invasive aspergillosis (IA) is a life-threatening fungal infection for immunocompromised hosts. It is, therefore, necessary to understand the immune pathways that control this infection. Although the primary infection site is the lungs, aspergillosis can disseminate to other organs through unknown mechanisms. Herein we have examined the *in vivo* role of various complement pathways as well as the complement receptors C3aR and C5aR1 during experimental systemic infection by *Aspergillus fumigatus*, the main species responsible for IA. We show that C3 knockout (C3^-/-^) mice are highly susceptible to systemic infection of *A. fumigatus*. Intriguingly, C4^-/-^ and factor B (FB)^-/-^ mice showed susceptibility similar to the wild-type mice, suggesting that either the complement pathways display functional redundancy during infection (i.e., one pathway compensates for the loss of the other), or complement is activated non-canonically by *A. fumigatus* protease. Our *in vitro* study substantiates the presence of C3 and C5 cleaving proteases in *A. fumigatus*. Examination of the importance of the terminal complement pathway employing C5^-/-^ and C5aR1^-/-^ mice reveals that it plays a vital role in the conidial clearance. This, in part, is due to the increased conidial uptake by phagocytes. Together, our data suggest that the complement deficiency enhances the susceptibility to systemic infection by *A. fumigatus*.

## Introduction

*Aspergillus fumigatus* is an opportunistic airborne fungal pathogen that causes invasive infection in immunocompromised individuals, such as those with hematological disorders, undergoing prolonged corticosteroid treatment (patients with organ transplantation), acquired-immunodeficiency syndrome, and autoimmune diseases (1). In immunocompetent hosts, inhaled *A. fumigatus* spores (conidia, the infective propagules) are cleared by the innate immune system. A significant body of knowledge is available on the clearance of conidia by the cellular immune system of the host (2–4). Still, the role of the humoral immune system, particularly the complement system, is poorly understood.

The complement system is a cascade of proteases that efficiently recognizes pathogens and kills them by lysis or tags them for removal by phagocytic cells. Besides, activation products of the complement system are capable of priming the acquired immune system by directly acting on T cell, B cell, and antigen-presenting cells (5–8). *A. fumigatus* is recognized by various complement activation pathway components such as C1q (9), MBL (10), ficolin-2 (11), and pentraxin-3 (PTX-3) (12). Therefore, it is not surprising that this pathogen, when incubated with serum *in vitro*, can activate all the three major (classical, alternative, and lectin) complement pathways (13–15). Initial studies on complement activation by various morphotypes of *A. fumigatus* showed that dormant conidia primarily activate the alternative pathway, while germinating morphotype activates the classical pathway (13, 14). A recent study showed that in normal serum, conidia initiate complement activation by the classical pathway, which then kicks off the alternative pathway amplification loop (16). However, when there is a lack of immunoglobulins (as in immunosuppressed hosts), the lectin pathway compensates for the classical pathway (17). Because *A. fumigatus* contains a thick cell wall, it is unlikely to be killed by the complement system by forming a membrane attack complex (MAC; C5b-9) in its cell membrane (13, 18). On the other hand, the complement system facilitates conidial clearance by promoting phagocytosis through conidial opsonization and/or by generating C5a, an anaphylatoxin (19). Indeed, the complement receptors CR3 and CR4 have been demonstrated to be important in the phagocytosis of opsonized *A. fumigatus* conidia (20).

To establish infection, *A. fumigatus* conidia employ a multitude of complement evasion mechanisms. For example, one of the cell wall components, melanin pigments, limits C3b deposition (21). Besides, conidia evade complement by recruiting host regulatory proteins and secreting proteases. The major soluble regulators of the complement system, such as factor H (FH), factor H-like protein (FHL-1), and C4b-binding protein (C4BP), have been shown to bind to the pathogen and regulate complement activation (22, 23). Two proteases associated with *A. fumigatus*, one released by its conidial morphotype (Mep1p) and another secreted by mycelia (Alp1p), have been shown to cleave major complement proteins C3, C4, and C5, and thereby inhibit phagocytosis (24, 25). Therefore, it is likely that these complement subversion strategies are enough to protect airborne *A. fumigatus* conidia from complement attack in the lung alveoli, where complement components are present in relatively lower amounts compared to blood. However, what role complement plays when the infection is disseminated and whether *A. fumigatus* can survive complement assault during systemic disease remain unclear. An earlier study demonstrated that C5-deficient mice (DBA/2N) display decreased resistance to systemic *A. fumigatus* infection compared to C5-sufficient outbred mice (CFW) (26). However, the genetic backgrounds of C5-deficient and -sufficient mice were different, posing difficulty in the data interpretation.

In the present study, we thus asked: (i) whether the complement system plays any protective role during systemic *A. fumigatus* infection, (ii) if yes, which complement pathways and/or components participate in this process, and (iii) what contribution, if any, do the anaphylatoxins C3a and C5a make in the host defense against systemic *A. fumigatus* infection? To decipher these questions, we employed a previously reported intravenous infection model to study disseminated aspergillosis (26, 27). Intravenous infection with *A. fumigatus* conidia in C3^-/-^ or C5^-/-^ mice resulted in significantly higher mortalities compared to that in wild-type mice, suggesting an essential role of the complement system against experimental systemic aspergillosis. *In vitro* study indicated that the conidia harbor C3 and C5 cleaving proteases. To dissect the role of anaphylatoxins during systemic aspergillosis, we performed studies using C3a receptor (C3aR^-/-^) and C5a receptor-1 knockout (C5aR1^-/-^) mice. The data suggested that C5aR1, but not C3aR signaling is required to mount a protective immune response against systemic *A. fumigatus* infection, which is partly due to enhanced uptake of conidia by phagocytes.

## Materials and methods

### *A. fumigatus* strain and growth conditions

A clinical isolate of *A. fumigatus* (28) was maintained on 2% malt-agar slant at ambient temperature. Conidia from 10-12 days old agar slants were harvested using 0.05% aqueous Tween-80 and passed through a filter (0.45 μm) to remove any hyphal fragments present, centrifuged (10,000 rpm, 10 min), resuspended in sterile water, freeze-dried, and stored at 4°C until use.

### Mice

The wild-type (BALB/c and C57BL/6) as well as the complement knockout mice (C3^-/-^ and C4^-/-^ on C57BL/6 background, C3aR^-/-^ and C5aR^-/-^ on BALB/c background, and B10.D2/oSnJ background for both C5-sufficient and C5-deficient) were purchased from the Jackson Laboratory, USA. C3^-/-^ mice on BALB/c background and factor B^-/-^ mice on C57BL/6 background were kind gifts from Prof. Marina Botto, Department of Medicine, Imperial College, London. All mice were bred and housed in a barrier-maintained and controlled animal facility of the National Centre for Cell Science (NCCS), Pune. All the animal experiments were approved by the Institutional animal ethics committee (IAEC) of NCCS, Pune (EAF/2016/B-207).

### *A. fumigatus* infection in mice

Wild-type (BALB/c and C57BL/6) and complement deficient mice (C3^-/-^, FB^-/-^, C4^-/-^, C5-sufficient, C5-deficient, C3aR^-/-^ and C5aR^-/-^) were used for the infection study. The mice were grouped (4-6 mice per cage) and marked on ear pinna by forming cuts. For infection, conidia were suspended in sterile PBS with 0.05% Tween 80 (PBS-T). A graded dose of conidial suspension (1x10^5^, 1x10^6^or 1x10^7^) in a volume of 100 µl was injected intravenously through the lateral tail vein. These mice were observed daily for survival and signs of illness such as head-tilt and swirling. The body weight of mice was monitored from the day of infection. Mice with body weight loss of ≥30% were sacrificed humanely and considered for mortality calculation.

### Complement proteins (C3 and C5), antibodies, sera, and plasma

Human C3 and C5 were obtained from Merck Millipore. Anti-mouse Ly-6G PE (Cat. No.:127614; clone:1A8), anti-mouse Ly-6C APC (Cat. No.:128008; clone:HK1.4), rat anti-mouse CD11b PE-Cy7 (Cat. No.:552850; clone.:M1/70) were purchased from BD Biosciences. Anti-mouse IgM (Cat. No.: 12-5890-82) was purchased from eBioscience. Blood was collected from infected mice by retro-orbital bleeding. For obtaining plasma, blood was collected in a microcentrifuge tube containing EDTA (final concentration, 20 mM) and centrifuged at 3000 rpm for 5 min at 4°C. For serum separation, blood collected in a microcentrifuge tube was kept at 37°C for 30 min and centrifuged at 3000 rpm for 5 min at 4°C. Plasma and sera were stored at -80°C until use.

### *In vivo* interaction of *A. fumigatus* conidia with neutrophils and monocytes

For measuring *in vivo* interaction of *A. fumigatus* conidia with neutrophils, eosinophils and monocyte, conidia were labeled with carboxyfluorescein diacetate succinimidyl ester (CFSE). In brief, dormant conidial suspension (1x10^7^ conidia/ml; 1 ml) in PBS-T was mixed with CFSE (2.5 μM) and incubated at 37°C in a water bath in the dark for 25 min, with vortexing every 5 min. To remove unbound CFSE, 300 µl of PBS-T was added to the suspension and centrifuged at 10,000 rpm. Conidia were then washed and suspended in PBS-T. CFSE-labeled conidia were analyzed with a FACS Calibur flow cytometer (BD Biosciences). The unstained conidia were used as a negative control for FACS analysis.

To examine the interaction of conidia with neutrophils/monocytes, CFSE-labeled conidia (1x10^6^;100 µl) were injected intravenously into WT and C3^-/-^ mice *via* the lateral tail vein. Next, about 0.5 ml of blood was collected in a sterile tube from each mouse by orbital bleeding at 5 min and 15 min post-infection and kept on ice. Blood was collected in 20 mM EDTA to avoid complement activation. To separate cells, blood was centrifuged at 3000 rpm, and RBCs were lysed by adding 4 ml of chilled lysis buffer (155 mM NH_4_Cl, 10 mM KHCO_3_, and 0.1 mM EDTA, pH 7.4) followed by incubation on ice for 4 min. The cell suspension was then mixed with 2 ml of chilled sterile PBS and centrifuged at 1800 rpm for 10 min at 4°C. The cells were washed twice with chilled sterile PBS to remove traces of lysis buffer. Hereafter, cells were suspended in 50 µl of sterile PBS and stained with fluorescently conjugated anti-CD11b, anti-Ly6G, and anti-Ly6C antibodies (0.5 µl of each antibody). The conidial interaction with the specific cell types was studied by flow cytometry, with a gating strategy: mature monocytes: Ly6G^-^ Ly6C^-^, immature monocytes: Ly6G^-^ Ly6C^+^, neutrophils: Ly6G^+^ Ly6C mid, and eosinophils: Ly6G^+^ Ly6C^-^ (**Supplemental Figure 1**). Data were analyzed by FlowJo software (TreeStar Inc.), and the total number of cells interacting with the conidia was plotted as a mean ± SD (three independent experiments).

### Complement C3 and C5 cleavage activity of *A. fumigatus*

To assess the complement C3 or C5 cleaving activity, 1x10^7^ conidia were incubated in a 50 µl of Tris buffer with human C3 or C5 (3 µg) for 30 min at 37°C. The reaction mixture was then centrifuged at 10,000 rpm for 5 min, and the supernatant was loaded on SDS-PAGE. The gel was stained with Coomassie blue to visualize the cleaved fragments. The protease activity was blocked by EDTA (10 mM), Chymostatin (100 µg/ml), or heat inactivation of conidia (95°C, 5 min). Conidial C3 cleavage capacity was also analyzed by Western blot at two different time-points: after 15 min and 30 min co-incubation of C3 with conidia. The C5a released upon C5 cleavage activity of conidia was measured by ELISA using the C5a DuoSet detection kit (R&D Systems).

### ELISA for mouse elastase, IL-6, and TNF-α and C5a measurements

Elastase, IL-6, and TNF-α were quantified using ELISA kits (R&D Systems). For C5a measurement, a 96-well plate (Greiner Bio-One) was coated with rat anti-mouse C5a antibodies (100 µl; 10 μg/ml in PBS; BD Biosciences Cat. No.: 558027) overnight at 4°C. The coated wells were blocked with 1% fetal bovine serum and 2% skimmed milk in PBS for 2 h at room temperature. After a wash with PBS-0.05% Tween-20, mice plasma (1:10 dilution) in blocking buffer was added to the wells; for the standard curve, different concentrations of C5a standard (BD Biosciences, Cat. No.: 51-9004473) was used. The plate was incubated for 2 h at room temperature and washed thrice with PBS-0.05% Tween-20. Bound C5a was measured by sequential incubations with biotinylated anti-mouse C5a antibody (1 µg/ml; BD Biosciences, Cat. No.: 558028), anti-avidin-HRP antibody (1:1000 diluted; Bio-Rad Cat. No. 170-6528) both suspended in blocking buffer, for 1 h, with PBS-T washes (thrice) between each step, followed by adding substrate (o-phenylenediamine dissolved in 0.1 M citrate buffer (pH 4.2) containing 1µl/ml of 30% H_2_O_2_;100 µl/well). After 20 min, the reaction was stopped by adding 3N H_2_SO_4_ (100 µl/well), and the color was read at 490 nm using a plate reader.

### Passive transfer of naïve sera

Naïve sera were obtained from WT (BALB/c) and C3^-/-^ (BALB/c) mice and incubated at 56°C for 30 min to inactivate complement. To determine the protective ability of the naïve mice sera from WT and C3^-/-^ mice against *A. fumigatus* infection, C3^-/-^ (BALB/c) mice were administered intraperitoneally with 250 µl of pooled serum on day -1 and day 4 post-infection. These mice were then challenged intravenously on day 0 with conidia (1x10^5^) suspended in PBS-T and examined for weight loss and mortality.

### Histopathology

For histopathology analysis, visceral organs (kidney, lung, spleen and liver) were harvested and immediately fixed in 10% neutral-buffered formalin (pH 7.4). The tissues were then processed by alcohol-xylene protocol, embedded in paraffin, and 5 μm size sections were cut using a microtome (Leica, Germany). The cut sections were stained with hematoxylin and eosin (H & E), and analyzed by observing under a light microscope.

### Periodic acid-Schiff staining

The sections used for histopathology analysis were also analyzed for PAS staining. For deparaffinization, the slide was flamed on the burner and placed in the xylene till all the wax was removed. Xylene was drained and the tissue section was hydrated by passing through decreasing concentrations of alcohol baths (100%, 90%, 80%, 70%) and water. The section was placed in a periodic acid solution (1%) for 5-10 minutes and thereafter the slide was washed with at least two changes of distilled water. Next, the slide was covered with Schiff’s reagent for 20-30 minutes followed by a rinse in running tap water for 10 minutes. Following this, the slide was covered with hematoxylin for 5 minutes and the section was dehydrated in increasing concentration of alcohols and placed in two xylene baths for clearing. Slides were mounted in DPX and analyzed under a light microscope.

### Statistical analysis

For *in vivo* studies, the percent survival of mice in each group was plotted as Kaplan-Meier plots and analyzed using the Log-Rank test (GraphPad Prism, GraphPad, San Diego, CA). Statistical analyses for *in vitro* assays were performed by Mann Whitney test (SigmaStat, Systat Software, Inc., San Jose, CA) for comparison of two groups and by one-way ANOVA (Tukey’s multiple comparison test, GraphPad prism) for comparison of multiple groups.

## Results

### Complement C3^-/-^ mice are highly susceptible to systemic *A. fumigatus* infection

**Figure 1:**
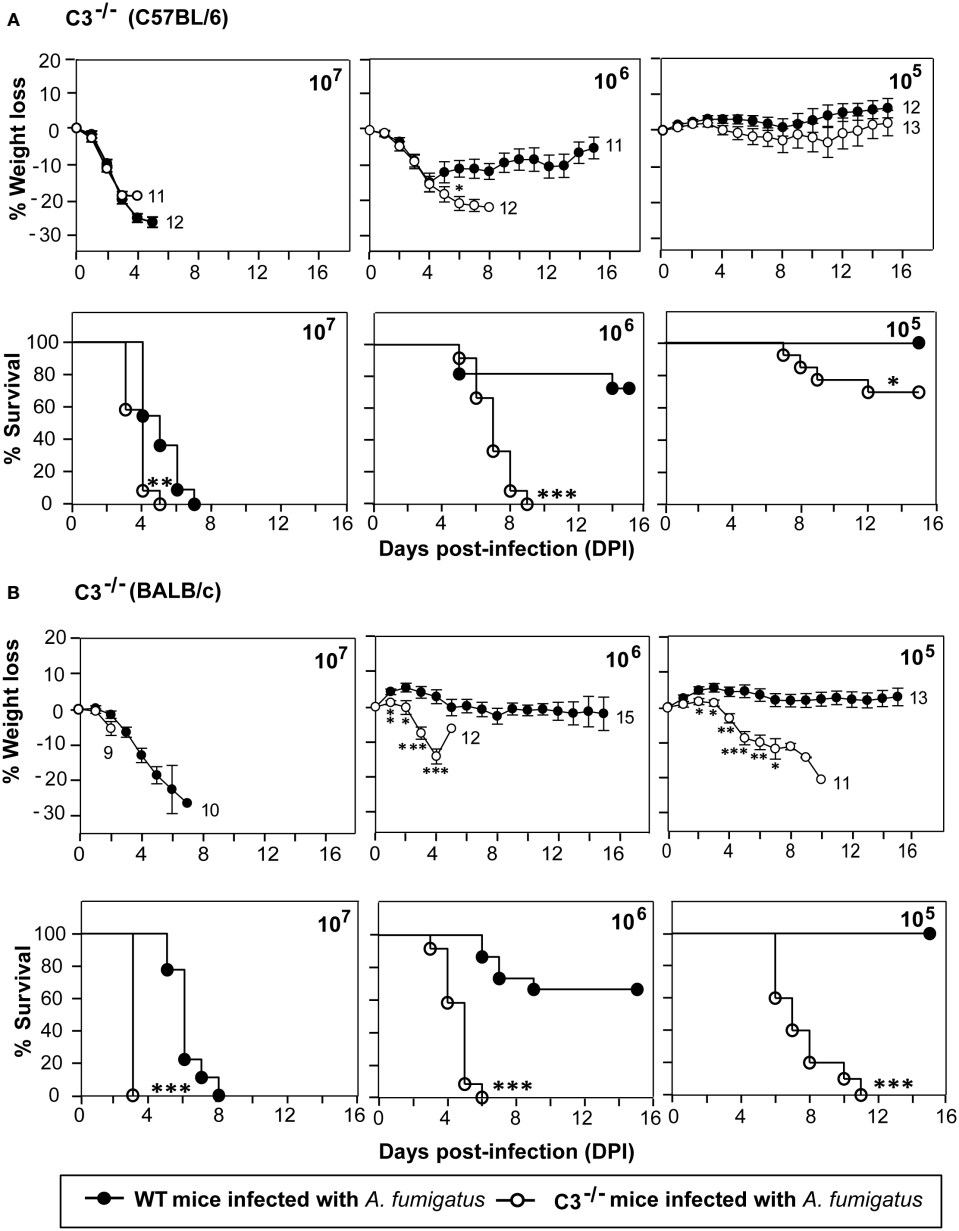
C3 deficiency enhances the susceptibility to systemic *A fumigatus* infection. Mice were intravenously infected with 1x10^5^, 1x10^6^, or 1x10^7^ conidia suspended in PBS-T. The percent body weight change and survival of mice after the infection was recorded daily for 15 days. **(A)** Percentage of body weight loss and survival in complement C3 deficient mice (C3^-/-^; C57BL/6 background) in comparison to the wild-type mice (WT; C57BL/6 background). The body weights were normalized to their initial body weights. The numbers at the end of the line depict the number of mice used in that group. The survival in C3^-/-^ mice group was significantly lower in comparison with WT mice group at 1x10^5^ and 1x10^6^ dose of conidial infection (% weight loss: *p < 0.05; % survival: ***p < 0.0001, **p < 0.001, *p < 0.01). **(B)** Percentage of body weight loss and survival in complement C3 deficient mice (C3^-/-^; BALB/c background) in comparison to the wild-type mice (WT; BALB/c background). Similar to C57BL/6 C3^-/-^ mice, the BALB/c C3^-/-^ mice also showed reduced survival at 1 x 10^5^ and 1 x 10^6^ dose of conidial infection in comparison to the WT mice (% weight loss: *p < 0.05, **p < 0.005, ***p < 0.0005; % survival: ***p < 0.0001). Results shown are mean ± SEM of three independent experiments.

C3 is the central component of the complement system. Its activation leads to the formation of C3b, which has been demonstrated to be essential for the *A. fumigatus* conidial opsonization and clearance by phagocytes (29). To explore the role of C3 during systemic *A. fumigatus* infection, we performed infection studies in C3^-/-^ mice (C57BL/6 background) and compared it with the wild-type (WT) mice. The mice were intravenously injected with high (1x10^7^), moderate (1x10^6^), and low (1x10^5^) doses of conidia (**Figure 1A**). At a high (1x10^7^) dose, both WT and C3^-/-^ mice failed to survive; however, in the C3^-/-^ mice group, 100% death occurred on the 5^th^ day post-infection (dpi) compared to the 7^th^ dpi in WT mice group. At a moderate (1x10^6^) dose, the C3^-/-^ mice showed 100% mortality, and WT mice showed only 30% mortality. Whereas at a low conidial dose (1x10^5^), C3^-/-^ mice group exhibited 30% mortality and WT mice showed 100% survival. Histopathological sections of different organs of conidia-challenged wild-type/C3-knockout mice were examined for fungal load (PAS staining) and infiltration of immune cells (H&E staining). The data indicated that in the C3-knockout mice, there is an increased fungal burden and inflammation, compared to the wild-type mice (**Supplemental Figures 2**-**4**), suggesting that the C3 deficiency contributes to both increased survival of the fungus and dysregulation of inflammation. Together, these results suggested that the C3^-/-^ mice are more susceptible to systemic *A. fumigatus* infection than the WT mice.

C57BL/6 mice preferentially develop a Th1 immune response, while BALB/c mice develop a Th2 immune response (27, 30). We thus sought to determine whether C3 deficiency in different genetic backgrounds affects the susceptibility to systemic infection by *A. fumigatus*. Therefore, as above, WT and C3^-/-^ mice, both on BALB/c background, were infected intravenously with various doses of *A. fumigatus* conidia. C3^-/-^ mice on BALB/c background were more susceptible to *A. fumigatus* systemic infection than that on a C57BL/6 background. Nevertheless, the survival patterns were essentially similar to those observed for C57BL/6 mice (**Figure 1B**). At the high dose of conidia (1x10^7^), all C3^-/-^mice succumbed to infection, but the mortality in WT mice was significantly delayed compared to C3^-/-^ mice. At the moderate dose (1x10^6^), all C3^-/-^ mice succumbed to infection compared to only 30% mortality in the WT mice group. Infection with a low conidial dose (1x10^5^) led to no mortality in the WT mice but 100% mortality in the C3^-/-^ mice group. Overall, these data demonstrate that complement component C3 is essentially required for the clearance of conidia from the systemic circulation regardless of the murine genetic background.

### Role of C4 and factor B in protection against systemic *A. fumigatus* infection

**Figure 2:**
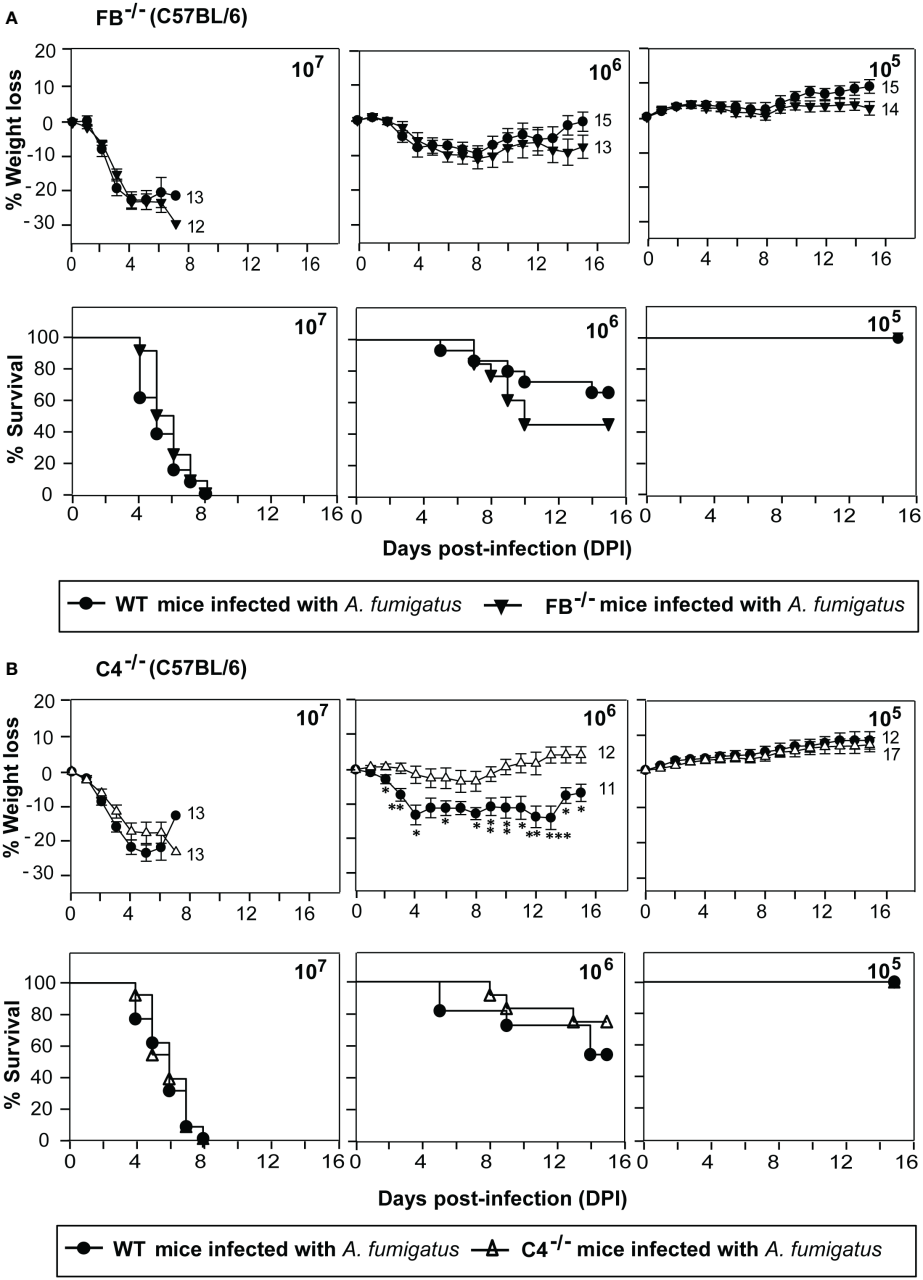
Susceptibility of factor B and C4 deficient mice to systemic *A fumigatus* infection. Mice were intravenously infected with indicated amounts of conidia (1x10^5^, 1x10^6^, or 1x10^7^) and observed for body weight loss and survival. **(A)** Percentage of body weight loss and survival in complement factor B deficient mice (FB^-/-^; C57BL/6 background) in comparison to the wild-type mice (WT; C57BL/6 background). There is no difference in the weight loss and survival of FB^-/-^mice compared to the WT mice. **(B)** Percentage of body weight loss and survival in complement C4 deficient mice (C4^-/-^; C57BL/6 background) in comparison to the wild-type mice (WT; C57BL/6 background). C4^-/-^ mice did not show more weight loss and reduced survival compared to the WT mice. (% weight loss: ***p < 0.0005; **p < 0.005 *p < 0.05). Results shown are mean ± SEM of three independent experiments.

Next, we examined the role of individual complement activation pathways during infection. Earlier *in vitro* studies have shown that *A. fumigatus* conidia and hyphae can activate all the three pathways of the complement system (13, 16, 17, 31), but which pathway is more critical during *in vivo* infection, is not known. We, therefore, intravenously infected factor B knockout (FB^-/-^) and complement C4 knockout (C4^-/-^) mice with conidia. The complement FB is a component of the alternative pathway; therefore, FB^-/-^ mice are deficient in the alternative pathway. C4, on the other hand, is a component of the classical and lectin pathways; hence, C4^-/-^ mice are deficient in these two pathways. Infection of FB^-/-^mice with conidia had no significant difference in survival and weight loss compared to WT mice (**Figure 2A**). C4^-/-^ mice also failed to show any difference in survival compared to WT mice (**Figure 2B**). This could be because of the redundancy, i.e., the lack of one pathway is compensated by the presence of the other, and therefore, both FB and C4 could be critical for the protection against *A. fumigatus* infection. It is also likely that during interaction with *A. fumigatus*, complement activation occurs primarily *via* a non-canonical pathway that does not require FB or C4.

**Figure 3:**
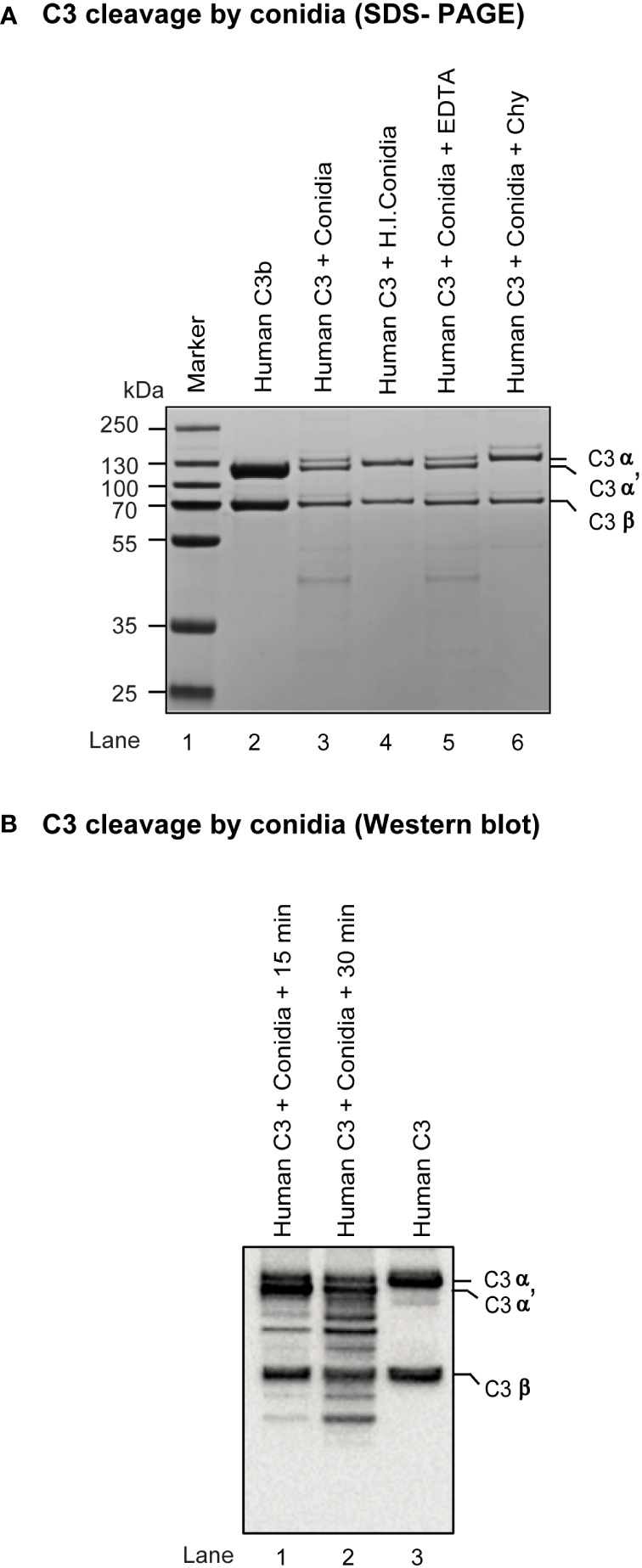
Cleavage of complement protein C3 by *A fumigatus* conidia. **(A)** Human C3 (3 µg) was incubated for 30 min at 37°C with 1x10^7^ conidia in a total reaction volume of 50 µl in Tris-buffer pH 7.4. Following incubation, the reaction mix was centrifuged at 10,000 rpm for 5 min. Supernatants were loaded on a 7.5% SDS-PAGE, and the cleaved fragments of C3 were visualized by staining the gel with Coomassie blue. Heat inactivated conidia: heat-inactivated by incubating it at 95°C for 5 min. Cleavage of C3 by *A fumigatus* conidia was performed in the presence of inhibitors: EDTA, (10 mM); Chy, Chymostatin (100 µg/ml). Conidia effectively cleaved C3, which could be inhibited by Chymostatin, but not by EDTA. The PAGE profile shown is a representative of three independent experiments. **(B)** C3 cleavage capacity of conidia was also examined by Western blot by incubating human C3 (3 µg) with 1x10^7^ conidia for 15-30 min at 37°C, collecting and subjecting the supernatant of the reaction mixture to SDS-PAGE (gradient gel, 4-12%), blotting separated protein bands on to nitrocellulose membrane, probing the membrane with anti-C3 antibody (Merci Millipore) and revealing the bands using ECL detection kit (Pierce); the experiment was repeated twice.

As *A. fumigatus* is known to encode many secreted proteases (32), we looked if conidia contain any surface protease capable of cleaving complement C3. Incubation of metabolically active conidia with human C3 resulted in the cleavage of C3 into a C3b-like fragment. Western blot indicated the C3b-like fragment formation and also captured further degradation products. Importantly, this cleavage could be blocked by chymostatin and heat-inactivation of conidia but not by EDTA, a metal chelator (**Figure 3**). The results suggested that conidia have protease(s) capable of activating complement. Further, it is not a metalloprotease, but likely a chymotrypsin-like serine/cysteine protease.

### Complement C5-deficient mice are more susceptible to systemic *A. fumigatus* infection

**Figure 4:**
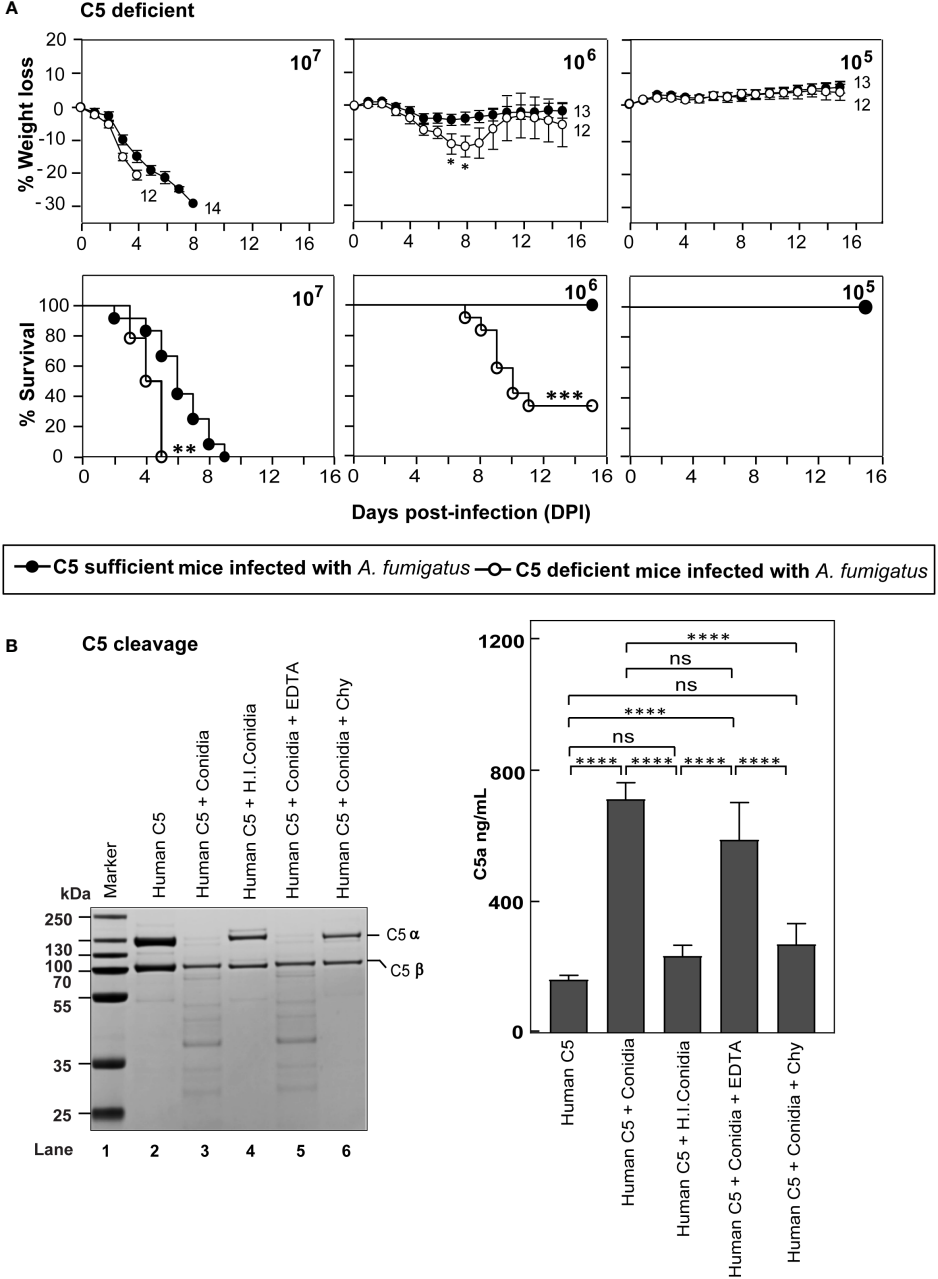
C5 deficiency enhances the susceptibility to systemic *A fumigatus* infection. **(A)** C5 sufficient (B10.D2/nSn) and C5 deficient (B10.D2/oSn) mice were infected intravenously with 1x10^5^, 1x10^6^ or 1x10^7^ conidia. The percent body weight loss and survival were monitored for 15 days. C5 deficient mice exhibited more susceptibility to *A fumigatus* infection than WT mice (***p < 0.0001; **p < 0.001 for survival and *p < 0.05 for weight loss). Results shown are mean ± SEM of three independent experiments. **(B)** Human C5 (3 µg) was incubated for 30 min at 37°C with 1x10^7^ conidia in a total reaction volume of 50 µl in Tris-buffer pH 7.4. Following incubation, the reaction mix was centrifuged at 10,000 rpm for 5 min. *Left panel*: Supernatants were loaded on a 12% SDS-PAGE, and the cleaved fragments of C5 were visualized by Coomassie blue staining. *Right panel*: Supernatants were tested for the presence of C5a by ELISA. Heat inactivated conidia: heat-inactivated by incubating it at 95°C for 5 min. Cleavage of C5 by *A fumigatus* conidia was performed in the presence of inhibitors: EDTA, (10 mM); Chy, Chymostatin (100 µg/ml). Conidia effectively cleaved C5, which could be inhibited by Chymostatin, but not by EDTA (ns – not significant, *p < 0.05, **p < 0.005, ***p < 0.0005). The statistical significance was evaluated by One way ANOVA, followed by Tukey’s multiple comparison test (****p < 0.01). ns, not significant.

Activation of terminal complement component C5 results in the generation of C5a and C5b. C5a is a potent immunomodulatory molecule that mediates the recruitment and activation of neutrophils and monocytes, which play a central role in the clearance of *A. fumigatus* conidia. C5b, on the other hand, initiates the formation of the terminal complement pathway and forms C5b-9 (MAC) on the pathogen leading to lysis, which is less likely to occur for *A. fumigatus* owing to its thick cell wall. Thus, if the terminal pathway is important, C5 deficiency is expected to exacerbate the disease. Therefore, the systemic infection was performed in C5-deficient and sufficient mice (**Figure 4A**). At a moderate dose (1x10^6^) of infection with conidia, the mortality in C5-deficient mice was 66%, whereas there was no mortality in C5-sufficient mice. With a high dose of conidia (1x10^7^), there was a significant delay in the death of C5-sufficient mice compared to C5-deficient mice. This result suggested that the complement C5 also plays an essential role against systemic *A. fumigatus* infection.

The susceptibility of C5-deficient mice to *A. fumigatus* infection led us to question whether conidia also have C5 degrading activity. To check this, we incubated human C5 with conidia (**Figure 4B**). Interestingly, upon incubating C5 with conidia, there was a degradation of the α-chain of C5; this C5 degrading activity was lost when conidia were subjected to prior heat-inactivation or in the presence of chymostatin, but not EDTA, suggesting that the conidial C5 degrading activity is also associated with a chymotrypsin-like serine/cysteine protease. Moreover, when we measured C5a released upon C5 incubation with conidia, there was a significant amount of C5a in the reaction mixture, which was found to decrease upon heat-inactivation of conidia or by the addition of chymostatin into the reaction mixture, but not upon EDTA addition (**Figure 4B**).

### C5a-C5aR axis plays a protective role during systemic *A. fumigatus* infection

**Figure 5:**
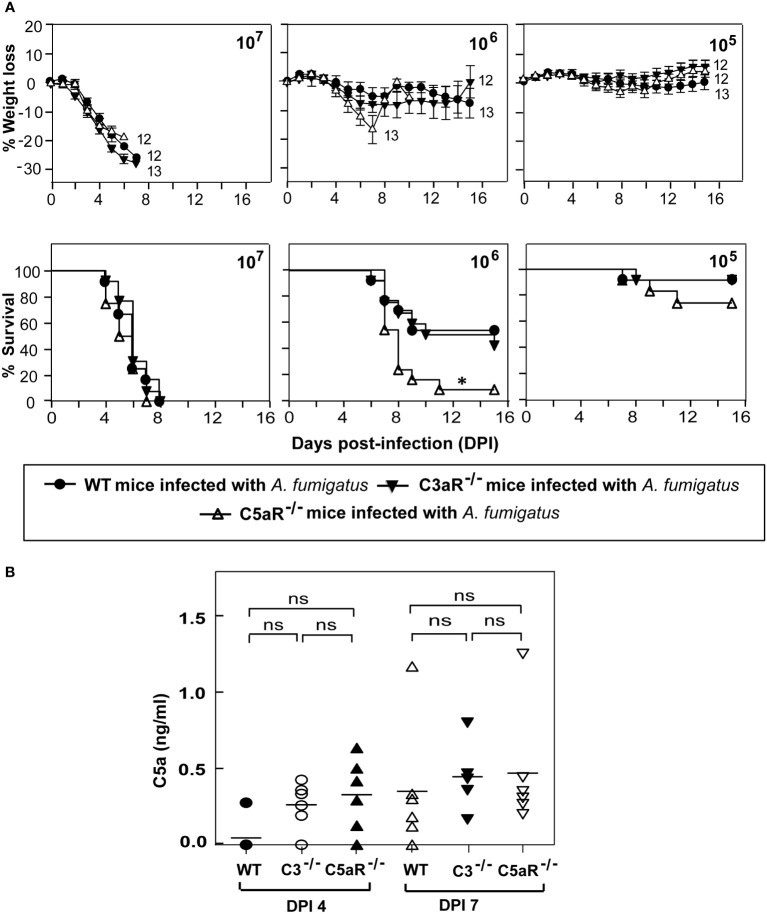
C5a receptor signaling is crucial for protecting against systemic *A fumigatus* infection. **(A)** Wild-type (WT), C3aR^-/-^ and C5aR^-/-^ mice (all on BALB/c background) were infected intravenously with 1x10^5^, 1x10^6^, or 1x10^7^ conidia and observed for percent body weight loss and survival till day 15 post-infection. The numbers depicted at the end of each line in the weight loss graph indicate the number of animals used in that group. The C5aR^-/-^ mice showed significantly higher mortality than the WT mice (*p < 0.01). Results represent the mean ± SEM of three independent experiments. **(B)** BALB/c WT, C3^-^/^-^ and C5aR^-^/^-^ mice were infected intravenously with 1x10^5^ conidia. Plasma samples from *A fumigatus* infected mice were obtained on day four and day seven post-infection. C5a levels were determined by ELISA. Results shown are mean concentrations ± SD obtained from six individual mice samples. The statistical significance was evaluated by One way ANOVA, followed by Tukey’s multiple comparison test. ns, not significant.

Activation of the complement system should result in the generation of two potent anaphylatoxins – C3a and C5a. These peptides modulate the functioning of various immune cells by binding to their receptors C3aR and C5aR1, respectively. Earlier reports have shown that the requirement of immune signaling *via* the C3a-C3aR and C5a-C5aR1 axes varies depending on the pathogen (33–36). To dissect the role of signaling through C3aR or C5aR1 axis in protection against systemic *A. fumigatus* infection, we infected C3aR^-/-^ and C5aR1^-/-^ mice with various doses of conidia (**Figure 5A**). At a moderate conidial dose (1x10^6^), the mortality in C5aR1^-/-^, but not C3aR^-/-^ mice, was significantly higher (~45%) than in the WT mice. At a low dose (1x10^5^) of infection, C5aR1^-/-^ mice showed comparatively more mortality than the WT mice, but the difference was not significant. It, therefore, can be concluded that the C5a-C5aR1 axis participates in immune protection against *A. fumigatus* infection. However, there is a caveat in this interpretation; for the ligand-receptor axis to work, the ligand must be available, and therefore, it is essential to assess the generation of C5a during the infection. Analysis of the serum of WT, C3^-/-^ and C5aR1^-/-^ mice infected with conidia showed that, although not at statistically different levels in different groups, the anaphylatoxin C5a was indeed generated during infection (**Figure 5B**).

### Passive administration of naïve sera from WT mice to C3^-/-^ mice extends their survival following systemic *A. fumigatus* infection

**Figure 6:**
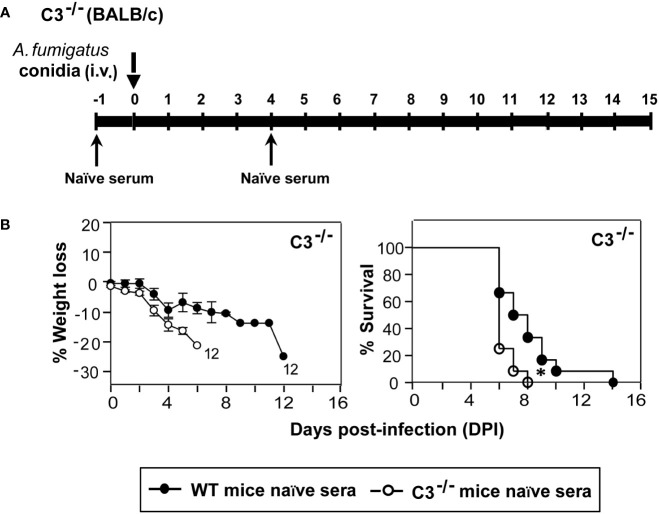
Natural antibodies aid in protection against systemic *A fumigatus* infection. **(A)** Diagrammatic depiction of the experimental setup. C3^-/-^ mice received 250 µl serum (i.p.) of naïve heat-inactivated sera from WT (BALB/c) or C3 deficient (C3^-/-^; BALB/c) at day -1 and day 4. These mice were challenged intravenously with 1x10^5^ conidia on day 0 and observed for body weight loss and mortality till the end of the experiment. **(B)** Body weight loss and survival of the infected complement C3^-/-^ mice that received heat-inactivated naïve sera either from WT or from C3^-/-^ mice. Passive transfer of naïve sera from WT mice extended the survival of infected C3^-/-^ mice (*p < 0.01). Results shown are mean ± SEM of two independent experiments.

Multiple reports show a convincing role of a natural reservoir of antibodies in protective immunity to bacteria, viruses, and *A. fumigatus* infection (37–39). Further, deficiency of complement receptor 2 (CR2) that binds to a C3 degradation product C3d has been linked to a reduced pool of peritoneal B1a cells, the primary source of natural antibodies (40). We thus tested the possibility of whether the lack of C3 results in decreased levels of natural antibodies, which in part is responsible for the higher susceptibility of C3^-/-^ mice to *A. fumigatus* infection. Here, we injected (i.p.) heat-inactivated naïve sera (to avoid any role of the complement system) from WT or C3^-/-^ mice to C3^-/-^ mice a day before the infection and at day 4 post-infection. These mice were then challenged with conidia and observed for body weight loss and mortality. The infected mice that received naïve sera from WT mice showed a significantly delayed mortality compared to mice that received the sera from C3^-/-^ mice (**Figure 6**). We, therefore, suggest that one of the ways by which complement C3 contributes to innate immunity against *A. fumigatus* is by enhancing the natural antibody pool.

### The contribution of the complement system in sequestering conidia during systemic *A. fumigatus* infection

**Figure 7:**
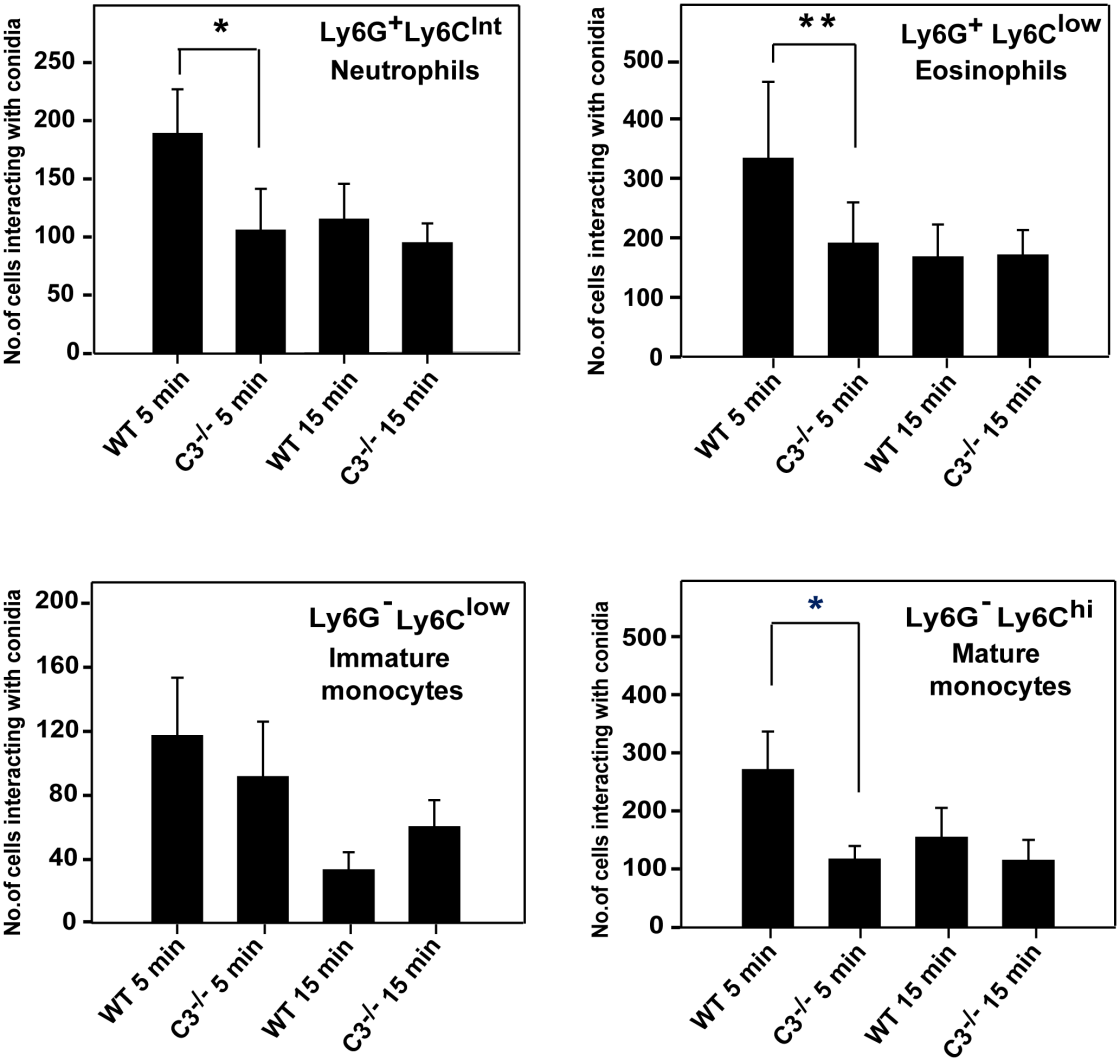
*In vivo* uptake of *A. fumigatus* conidia by monocyte subsets, eosinophils, and neutrophils. *A. fumigatus* conidia 1x10^6^ labelled with CFSE were injected intravenously in wild-type (WT) and C3^-/-^ mice (both on BALB/c background), and blood was collected from infected mice from the retro-orbital sinus at 5 min and 15 min post-infection. Following RBC lysis, cells were stained for neutrophils (Ly6G^+^/Ly6C^Int^), eosinophils (Ly6G^+^/Ly6C^low^), and monocyte subsets (Ly6G^-^/Ly6C^low^ and Ly6G^-^/Ly6C^hi^) with fluorescent antibody and uptake/interaction of conidia was examined by FACS. Results represent the mean ± SD of three independent experiments. Immune cells from WT mice showed more interaction with conidia in 5 min than the cells from C3^-/-^ mice. The statistical significance in complement deficient mice was evaluated by Mann Whitney test (**p < 0.01 *p < 0.05).

Another plausible reason for the higher susceptibility of C3^-/-^ mice to systemic *A. fumigatus* infection could be the lack of conidial opsonization by C3b, affecting their uptake by neutrophils, monocytes, and eosinophils *via* complement receptors, and thereby reducing conidial clearance. To test this possibility, WT and C3^-/-^ mice were intravenously infected with CFSE-labeled conidia, and blood lymphocytes were analyzed for the uptake of labeled conidia. As speculated, immediately after infection (5 min post-infection), neutrophils (Ly6G^+^Ly6C^Int^), eosinophils (Ly6G^+^Ly6C^low^), and monocytes (Ly6G^-^Ly6C^low^ and Ly6G^-^Ly6C^hi^) from WT mice engulfed a significantly higher number of conidia compared to that from C3^-/-^ mice (**Figure 7**). The uptake of conidia reduced with time. There was no difference in the uptake between WT and C3^-/-^ mice following 15 min post-infection (**Figure 7**), possibly due to other modes of phagocytosis. Nevertheless, these data imply that C3 opsonization affects conidial uptake by monocytes, neutrophils, and eosinophils *in vivo*, and this in part is likely responsible for reduced immune clearance of conidia in C3^-/-^ mice.

### Expression of other effectors during systemic *A. fumigatus* infection in C3^-/-^and C5aR1^-/-^ mice

**Figure 8:**
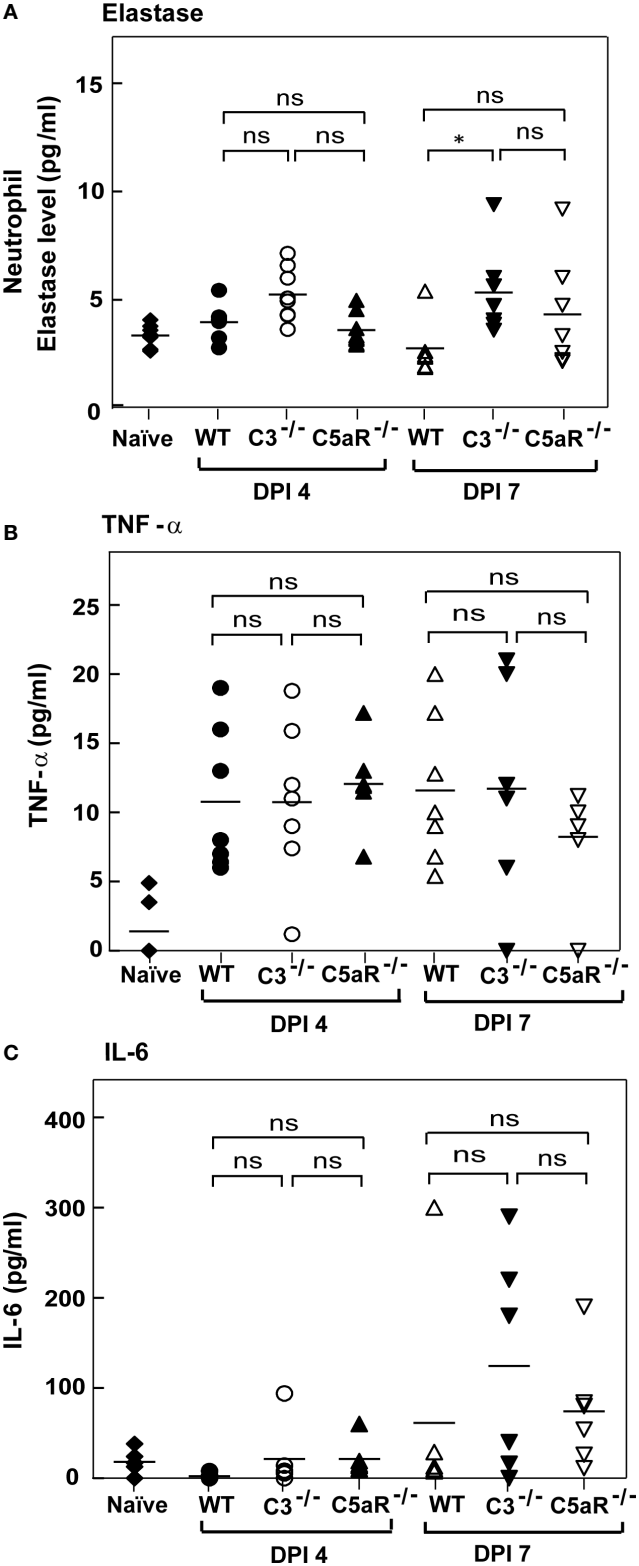
Complement deficient mice with systemic *Aspergillus* infection show elastase, TNF-alpha and IL-6 comparable to wild-type mice. **(A)** BALB/c WT, C3^-/-^ and C5aR^-/-^ mice were infected intravenously with 1x10^5^ conidia. Plasma samples from *A fumigatus* infected mice were obtained on day four and day seven post-infection. Levels of elastase in the infected samples were determined by ELISAs and compared with the WT mice infected with *A fumigatus* conidia. Results shown are mean concentrations ± SD obtained from six individual mice samples. The statistical significance in complement deficient mice was evaluated by Tukey’s multiple comparison test (*p < 0.05). **(B, C)** BALB/c WT, C3^-/-^ and C5aR^-/-^ mice were infected intravenously with 1x10^5^ conidia. Serum samples from *A fumigatus* infected mice were obtained on day four and day seven post-infections. No significant difference was observed with the TNF-α levels. IL-6 levels were increased in C3^-/-^ and C5aR^-/-^ mice on 7 post-infection day compared with infected WT mice, but the difference is not significant. The statistical significance was evaluated by One way ANOVA, followed by Tukey’s multiple comparison test (ns, not significant).

It is clear from our data that complement deficiency results in heightened susceptibility to *A. fumigatus* infection. We thus asked whether increased susceptibility of C3^-/-^ and C5aR1^-/-^ mice to *A. fumigatus* infection is also due to reduced generation of other effectors in these mice known to control *A. fumigatus* infection. For example, deficiency in elastase released by neutrophils has been illustrated to delay the clearance of *A. fumigatus* (41). Nonetheless, estimation and comparison of neutrophil elastase levels between WT, C3^-/-^ and C5aR1^-/-^mice (all on BALB/c background) infected with *A. fumigatus* conidia showed that all the three groups of mice produce similar levels of elastase (**Figure 8A**). TNF-α and IL-6 are the cytokines involved in the defense against *A. fumigatus*, and both TNF-α^-/-^ and IL-6^-/-^ mice display increased susceptibility to *Aspergillus* infection (42–44). However, the quantitation of TNF-α levels in WT and C3^-/-^or C5aR1^-/-^ mice in our study illustrated that its level increased after infection (day 4) and remained high even on day 7 (**Figure 8B**). The IL-6 levels also increased with the time of infection but did not vary between WT and complement knockout (C3^-/-^ and C5aR1^-/-^) mice groups (**Figure 8C**). We, therefore, conclude that the increased mortality in the C3^-/-^ and C5aR1^-/-^ mice are not due to generalized failure in generating some of the salient effectors against *A. fumigatus*.

## Discussion

A high mortality rate and a limited number of drugs available for treating invasive aspergillosis solicit increased knowledge on the antifungal immune response that controls *A. fumigatus* infection. The innate immune system forms the first barrier against pathogens, wherein the complement system plays a central role. Earlier *in vitro* studies have established the role of complement components and complement receptors in detecting and eliminating *A. fumigatus* conidia. However, the contribution of these entities during *in vivo* infection is not fully understood. In this study, we assessed the role of the complement system in a mouse model of experimental systemic aspergillosis (26, 27). Our *in vivo* model mimics the scenario of systemic aspergillosis, where the spores cross the lung barrier to establish systemic infection. In this situation, the complement system is likely to have a more prominent role, as complement proteins are readily available in circulation. In agreement, our data show that the complement system plays an indispensable role during systemic aspergillosis. We demonstrate that the deficiencies of complement component C3 enhance lethality due to decreased uptake-clearance of conidia by the phagocytes. We also show that the C5a-C5aR1 axis plays a critical role in resistance against *A. fumigatus*, as C5-deficiency increased susceptibility to systemic *A. fumigatus* infection.

Earlier *in vitro* studies have shown that all the morphotypes of *A. fumigatus* are capable of activating the complement system. Dormant conidia primarily activate the alternative pathway, while hyphal morphotypes activate the classical and lectin pathways (13, 14). Further, it has also been documented *in vitro* that the activation results in the deposition of C3b on the *A. fumigatus* conidial surface, which facilitate conidial phagocytosis when recognized by the complement receptors CR3 and/or CR4 on the immune cells (20). Whether complement plays a role in protection against systemic aspergillosis has not yet been adequately tested. An earlier study noted a higher degree of lethality in a systemic model of aspergillosis in DBA/2N mice lacking complement C5, compared to complement sufficient outbred CFW mice (26). Activation of C3 is the central step in the complement cascade. Therefore, to investigate the *in vivo* role of intact complement during systemic infection, we utilized C3^-/-^ mice. We also examined if C3 deficiency on different genetic backgrounds alters the susceptibility to aspergillosis, particularly on Th1-biased C57BL/6 and Th2-biased BALB/c mice. We observed that the C3 deficiency enhances the susceptibility to experimental systemic aspergillosis on both genetic backgrounds. However, the mortality was higher on BALB/c background, which is expected due to the dominance of Th2 response. These results are consistent with the observation that during aspergillosis, administration of Th1 cytokines results in a better outcome (45). It is relevant to point out that an earlier study showed that C3 deficiency enhances susceptibility to systemic *Candida* infection in mice (46).

Pathogens are known to activate single or multiple complement pathways, offering protection *via* complement-mediated mechanisms. For example, the classical pathway activation is required for effective immune defense against *Streptococcus pneumoniae* infection (47) and polymicrobial peritonitis (48), while the lectin pathway is a major defense against pneumococcal infection (49). However, in some pathogens, multiple pathways synergize to provide effective protection (33, 50). To determine the contribution of each of the pathways during systemic *A. fumigatus* infection, we infected FB^-/-^ (deficient in the alternative pathway) and C4^-/-^ (deficient in the classical/lectin pathways) mice. To our surprise, we observed no difference in the mortality of these mice compared to that in the WT mice. The data thus suggested a possibility of activation of complement without the involvement of C4 and C2. The existence of such a pathway has been known for over four decades now (51) and has been demonstrated in the classical (52, 53) and lectin (54) pathways. Such activation, however, requires an intact alternative complement pathway. Of note, *Aspergillus* conidia have been shown to activate complement by mannan-binding lectin (MBL) C2 bypass mechanism (15). MBL is a *C*-type lectin important for activating the lectin pathway. In an earlier study, upon intravenous infection with *A. fumigatus* conidia, both MBL knockout (both A and C genes deletion on B6.129S4 background) and WT mice (C57BL/6) showed comparable mortality profiles at higher doses of conidia. At the lowest dose, though the death of knockout mice was slower, the survival of MBL^-/-^ mice was not significantly different from that of WT mice (55).

Since C4/C2 bypass pathways are known to involve the alternative pathway and we did not observe increased mortality in FB^-/-^ mice infected with *A. fumigatus*, we considered a possibility of direct activation of complement by the pathogen-associated protease. To probe this further, we looked for the presence of a conidial protease that can cleave C3 and C5. We indeed found the presence of such a protease associated with conidia that can cleave C3 into a C3b-like fragment and C5 into smaller fragments generating C5a. Apparently, it looks like conidia facilitate their clearance by converting C3 into C3b-like fragment that leads to opsonization-mediated conidial phagocytosis and C5 into C5a that mobilize phagocytes. The interesting question is, then, how does *A. fumigatus* balances its survival from complement destruction? It should be noted that *A. fumigatus* produces proteases like Mep1p and Alp1p that are capable of inactivating C3 and C5 and their activation products (C3a and C5a). These proteases are released into the medium containing albumin/collagen (24, 25), suggesting that *A. fumigatus* conidia escaping from phagocytotic clearance (as in immunosuppressed condition) are well equipped to protect themself in the lung environment where the complement concentration is relatively lower compared to that in the blood.

The late complement components (C5-C9) are essential to clear most infections. In the context of fungal infection, complement C5 has been identified as the dominant gene responsible for altering the susceptibility to systemic *Candida* infection (56). The late complement components provide protection by two mechanisms: i) by forming a membrane attack complex (MAC; C5b-9) that kills pathogens by pore formation, and ii) as mentioned above, by generating C5a anaphylatoxin that causes the mobilization of neutrophils (57) and monocytes to the site of infection for clearance. Besides, C5a also stimulates neutrophils to produce toxic oxygen-derived free radicals and monocytes to produce inflammatory cytokines. Our infection studies in congenic C5-sufficient and C5-deficient mice indicated that C5 deficiency exhibits significantly higher mortality following systemic *A. fumigatus* infection. It is therefore clear that C5 contributes to host resistance to *A. fumigatus*. The next obvious question was how it contributes to the host resistance – owing to MAC-mediated lysis or C5a-mediated immune potentiation. The thick cell wall of conidia/hyphae is expected to block the MAC-mediated lysis of *A. fumigatus* (18, 58); therefore, it is unlikely that C5 contributes *via* MAC formation. We thus asked, does the C5a-C5aR1 axis play a part in immune protection? We also evaluated the participation of the C3a-C3aR axis, which has been reported to inhibit neutrophil mobilization (59). Survival analysis of the infected mice revealed enhanced mortality in C5aR1^-/-^, but not C3aR^-/-^ mice compared to the WT mice. We thus conclude that C5-mediated protection against experimental systemic aspergillosis is owing to C5a-C5aR signaling. Having said that, we would like to point out that the severity of infection in C5aR1^-/-^ mice was comparatively less than in C3^-/-^ mice (see mortality at 1x10^5^ doses of conidia in **Figure 1B** and **Figure 5**; both mice on BALB/c background), and therefore signaling through the C5aR1 receptor is only partially responsible for providing complement-mediated protection.

The upregulation of Th1 and proinflammatory cytokines such as IFN-γ, TNF-α, IL-6, IL-12, GM-CSF, IL-1, and IL-18 are associated with a better prognosis of systemic aspergillosis (45). Nagai et al. (42) showed that BALB/c mice upon intravenous *A. fumigatus* infection showed reduced death from 40-80% to zero when treated with IFN-γ or TNF-α. Camargo et al. (44), on the other hand, showed that the levels of IL-6 were higher after *in vitro* culturing the peripheral blood mononuclear cell from the healthy and hematological controls but not from the patients with invasive aspergillosis implicating the importance of the IL-6/STAT3 pathway in protective immunity against *Aspergillus*. Coherent with this, Cenci et al. (43) reported that IL-6^-/-^ mice were more susceptible than WT mice to invasive pulmonary aspergillosis. Intriguingly, the Th1 cytokines IL-6 and TNF-α have a direct link with C5a in that it induces the synthesis of these cytokines (60–63). We thus sought to determine whether the serum levels of these cytokines differ in infected C3^-/-^ and C5aR1^-/-^ mice compared to that in infected wild-type mice. However, the levels of these cytokines were comparable in knockout and wild-type mice, suggesting higher mortality in complement knockout mice is not due to decreased production of these protective cytokines. Next, we examined the uptake of the conidia by phagocytes. Our data revealed that the rate of association of conidia with the phagocytes is significantly higher in WT mice compared to C3^-/-^ mice. Therefore, it appears that the complement system contributes to the clearance of conidia during systemic *A. fumigatus* infection.

The complement receptors CR1/CR2, which bind to C3-derived ligands, have been shown to shape the natural antibody (nAbs) repertoire (64). These polyreactive low-affinity nAbs contribute to the defense against various infections, including that caused by *A. fumigatus* (37, 65). It is believed that these nAbs mediate protection by enhancing complement activation and deposition, promoting phagocytosis and antibody-dependent cellular cytotoxicity. Moreover, they inhibit fungal growth, adherence, germination, and biofilm formation (66). We thus investigated whether the C3 deficiency results in a decreased nAbs pool, which in part causes higher lethality in C3^-/-^ mice following systemic *A. fumigatus* infection. To test this, we passively transferred the naïve heat-inactivated sera from WT or C3^-/-^ mice to C3^-/-^ mice and challenged them with the conidia a day after the transfer. Although the WT mice sera could not rescue the infected mice from lethal infection, it prolonged survival compared to C3^-/-^ mice sera. Consequently, the decreased levels of natural antibodies in C3^-/-^ mice might be another reason responsible for their higher susceptibility to *Aspergillus* infection.

In summary, our data uncovered the importance of the complement system, an integral part of the humoral immune system of the host, during experimental systemic aspergillosis. Based on our data, we propose the following mechanism for complement-mediated protection against *A. fumigatus* infection: (i) *A. fumigatus* conidia are endowed with a protease, which can activate the complement system by a non-canonical pathway, (ii) C3b opsonization that occurs as a result of complement activation enhances the rate of phagocytosis *via* complement receptors CR3/CR4 (67), and iii) C5a generated owing to complement activation triggers the activation of phagocytic cells *via* C5aR1, and this results in migration and tissue penetration of phagocytes for fungal clearance. The effect of C5a, however, is not owing to increased synthesis of the cytokines IL-6 and TNF-α.

## Data availability statement

The original contributions presented in the study are included in the article/**Supplementary Material**. Further inquiries can be directed to the corresponding authors.

## Ethics statement

The animal study was reviewed and approved by Institutional animal ethics committee (IAEC) of NCCS, Pune.

## Author contributions

AS, RS, VA, JP, and GL designed and planned this study. RS, SW, and HM performed different experiments. RS and AS analyzed the data. VA, SW, TM, and AS contributed the reagents, materials, and/or analysis tools. RS and AS drafted the manuscript. All authors contributed to the article and approved the submitted version.

## Funding

AS is a J.C. Bose National Fellow. His lab is supported by the Department of Biotechnology, India, Project Grant BT/PR28506/MED/29/1307/2018. VA was supported by FUNHYDRO (ANR-16S-CE110020-01) and FUNPOLYVAC (ANR-21-CE17-0032) grants. SW was supported by Pasteur-Roux-Cantarini postdoctoral fellowship.

## Acknowledgments

We thank Mr. Sandip Sonar (National Centre for Cell Science, Pune) for his help during intravenous injections and Dr. B. Ramanamurthy for providing mice. A part of this work was done in partial fulfilment of the Ph.D. thesis of RS submitted to Dr. D.Y. Patil Vidyapeeth, Pune.

## Conflict of interest

The authors declare that the research was conducted in the absence of any commercial or financial relationships that could be construed as a potential conflict of interest.

## Publisher’s note

All claims expressed in this article are solely those of the authors and do not necessarily represent those of their affiliated organizations, or those of the publisher, the editors and the reviewers. Any product that may be evaluated in this article, or claim that may be made by its manufacturer, is not guaranteed or endorsed by the publisher.

## Supplementary material

Supplementary Figure 1 Experimental strategy for *in vivo* uptake of conidia by monocyte subsets, eosinophils, and neutrophils.

Supplementary Figure 2 Histopathological changes in the sections of lung and spleen tissues of mice challenged with *A. fumigatus* at day 7 post-infection. WT (left side) and C3^-^/^-^ (right side) mice were infected intravenously with 1 X 10^5^ dose of *A. fumigatus* conidia and sacrificed at day 7 post-infection. The lungs and spleen were harvested for histopathology. Sections were stained with H & E and are representative of each group (n = 4 to 5, in each group). **(A)** Typical histopathological changes for lung include Emphysema (), Peri-vascular aggregation of MNCs (**>**), acute inflammatory status (). B = bronchi. **(B)** Typical histopathological changes for the spleen include: hyperplasia of lymphoid tissue in white pulp ().

Supplementary Figure 3 Histopathological changes in the sections of liver and kidney tissues of mice challenged with *A. fumigatus* at day 7 post-infection. WT (left side) and C3^-^/^-^ (right side) mice were infected intravenously with 1 X 10^5^ dose of *A. fumigatus* conidia and sacrificed at day 7 post-infection. The liver and kidneys were harvested for histopathology. Sections were stained with H & E and are representative of each group (n = 4 to 5, in each group). a) Examination of kidney section on day 7 showed more promptly focal cellular swelling of renal tubular epithelium in cortico-medullary region in C3^-/-^ mice than WT mice. The changes include cellular swelling of renal tubular epithelium (), and vascular congestion (VC). b) Hepatocyte with intact cellular borders and nucleus was detected in the liver section for WT and C3^-^/^-^ mice. The nuclear swelling was graded high for liver histopathology in C3^-^/^-^ mice compared to WT mice liver. The changes include: cellular swelling of hepatocytes (), granular degeneration (), nuclear swelling of hepatocytes () and vascular congestion (VC).

Supplementary Figure 4 Periodic acid-Schiff (PAS) staining of the sections of lung, spleen, liver, and kidney tissues of mice challenged with *A. fumigatus* at day 7 post-infection. WT (upper panel) and C3^-/-^ (lower panel) mice were infected intravenously with 1 X 10^5^ dose of *A. fumigatus conidia*, sacrificed at day 7 post-infection. The lungs, spleen, liver and kidneys were harvested for histopathology. Representative image of PAS-staining for each group (n = 4 to 5, in each group) for **(A)** lung, **(B)** spleen, **(C)** kidney, and **(D)** liver. The sections showed mass of hyphae (), hyphae (), area of very high density hyphae () and branched hyphae ().
