# Supplementary figures and images for: Protective role of host complement system in Aspergillus fumigatus infection
Source: Front Immunol. 2022 Sep 23;13:978152. doi: 10.3389/fimmu.2022.978152 (PMC9539816; doi:10.3389/fimmu.2022.978152)

Supplemental Figure 1

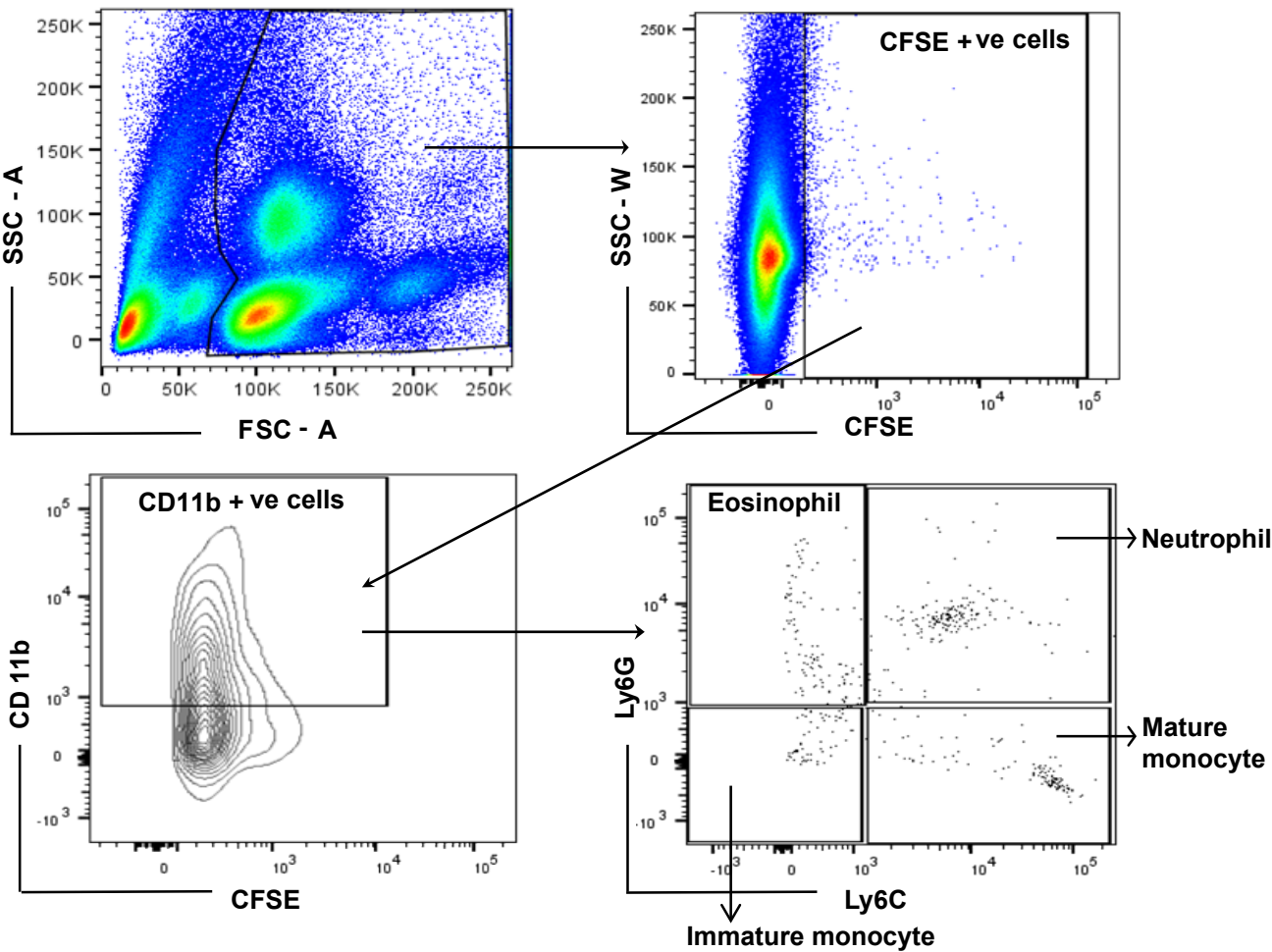

Supplement: Supplementary Figure 1 — Experimental strategy for in vivo uptake of conidia by monocyte subsets, eosinophils, and neutrophils. [file DataSheet_1.pdf]

## Supplemental Figure 2

a : Lung day 7 post -infection

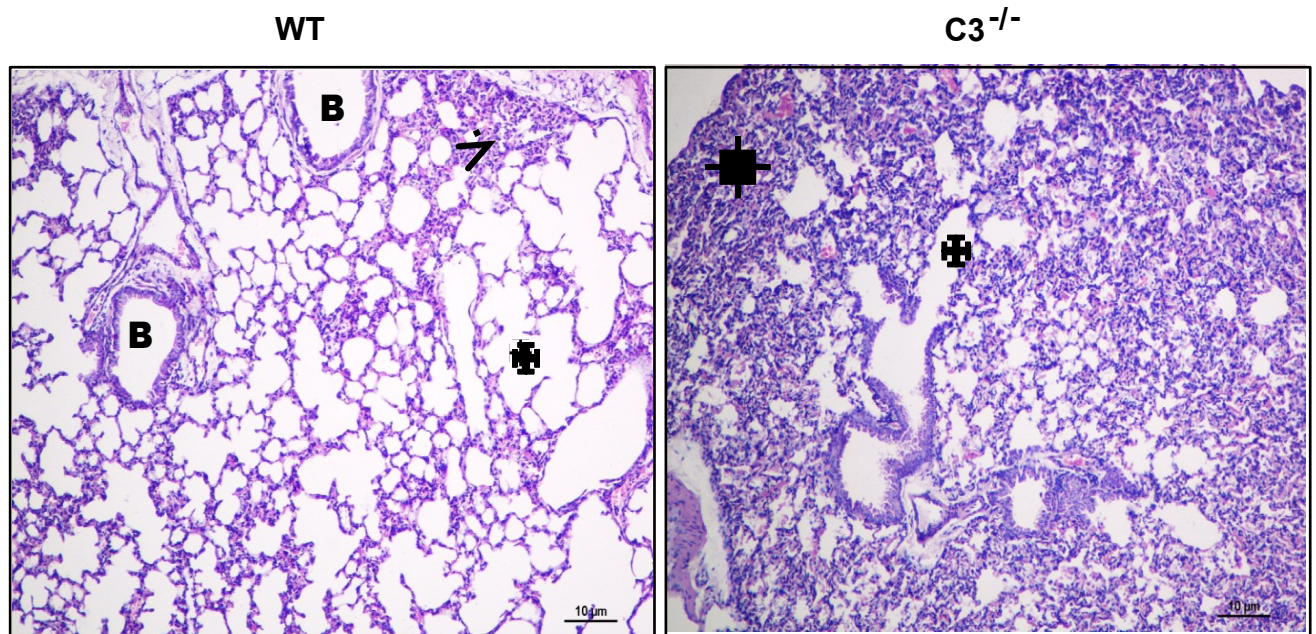

b : Spleen day 7 post -infection

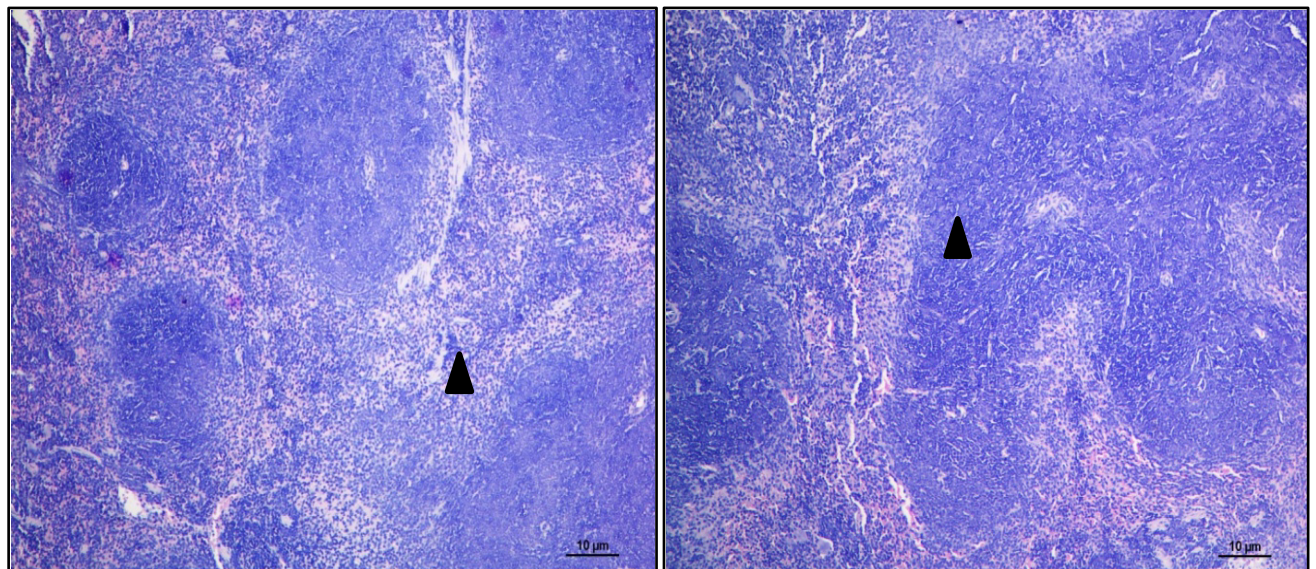

Supplement: Supplementary Figure 2 — Histopathological changes in the sections of lung and spleen tissues of mice challenged with A. fumigatus at day 7 post-infection. WT (left side) and C3-/- (right side) mice were infected intravenously with 1 X 105 dose of A. fumigatus conidia and sacrificed at day 7 post-infection. The lungs and spleen were harvested for histopathology. Sections were stained with H & E and are representative of each group (n = 4 to 5, in each group). (A) Typical histopathological changes for lung include Emphysema (), Peri-vascular aggregation of MNCs (>), acute inflammatory status (). B = bronchi. (B) Typical histopathological changes for the spleen include: hyperplasia of lymphoid tissue in white pulp (). [file DataSheet_2.pdf]

## Supplemental Figure 3

a : Kidney day 7 post -infection

WT

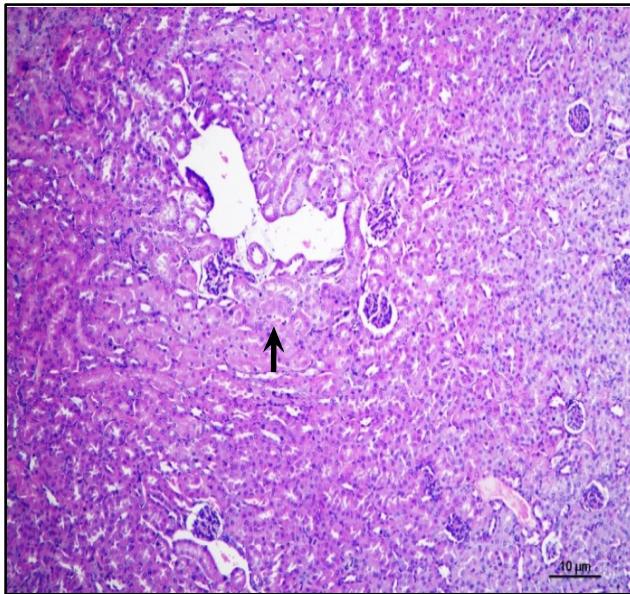

C3<sup>-/-</sup>

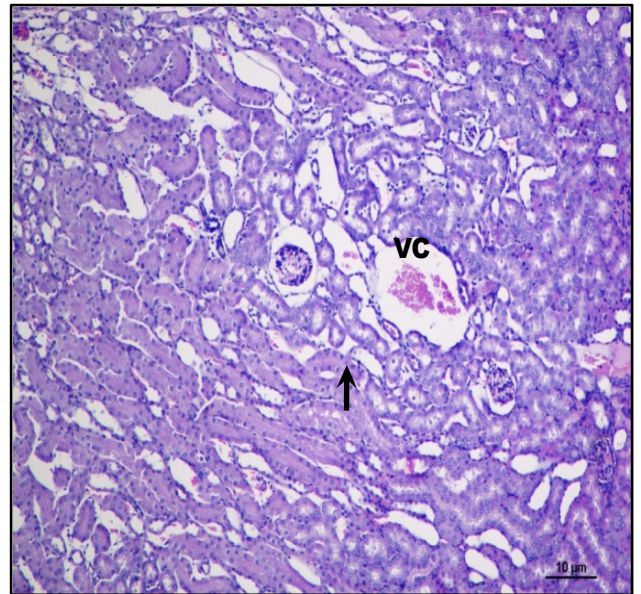

b : Liver day 7 post -infection

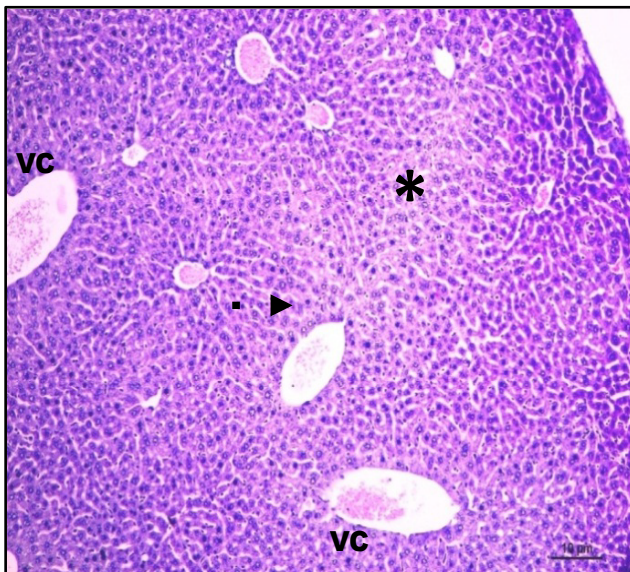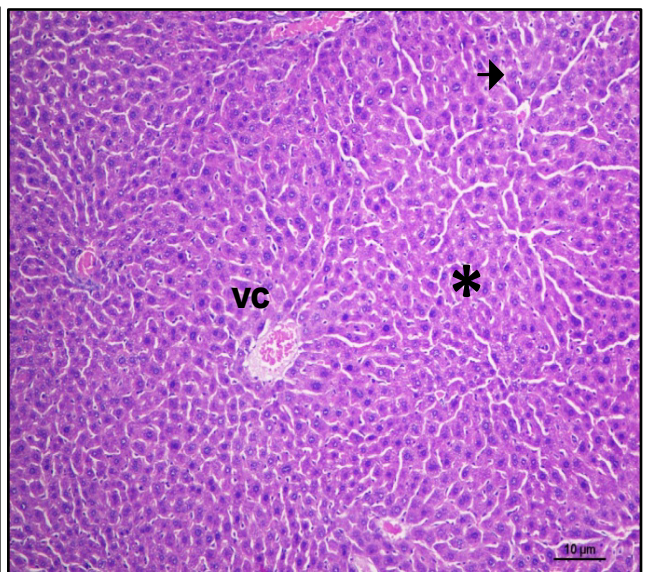

Supplement: Supplementary Figure 3 — Histopathological changes in the sections of liver and kidney tissues of mice challenged with A. fumigatus at day 7 post-infection. WT (left side) and C3-/- (right side) mice were infected intravenously with 1 X 105 dose of A. fumigatus conidia and sacrificed at day 7 post-infection. The liver and kidneys were harvested for histopathology. Sections were stained with H & E and are representative of each group (n = 4 to 5, in each group). a) Examination of kidney section on day 7 showed more promptly focal cellular swelling of renal tubular epithelium in cortico-medullary region in C3-/- mice than WT mice. The changes include cellular swelling of renal tubular epithelium (), and vascular congestion (VC). b) Hepatocyte with intact cellular borders and nucleus was detected in the liver section for WT and C3-/- mice. The nuclear swelling was graded high for liver histopathology in C3-/- mice compared to WT mice liver. The changes include: cellular swelling of hepatocytes (), granular degeneration (), nuclear swelling of hepatocytes () and vascular congestion (VC). [file DataSheet_3.pdf]

## Supplemental Figure 4

a : Lung day 7 post -infection

WT

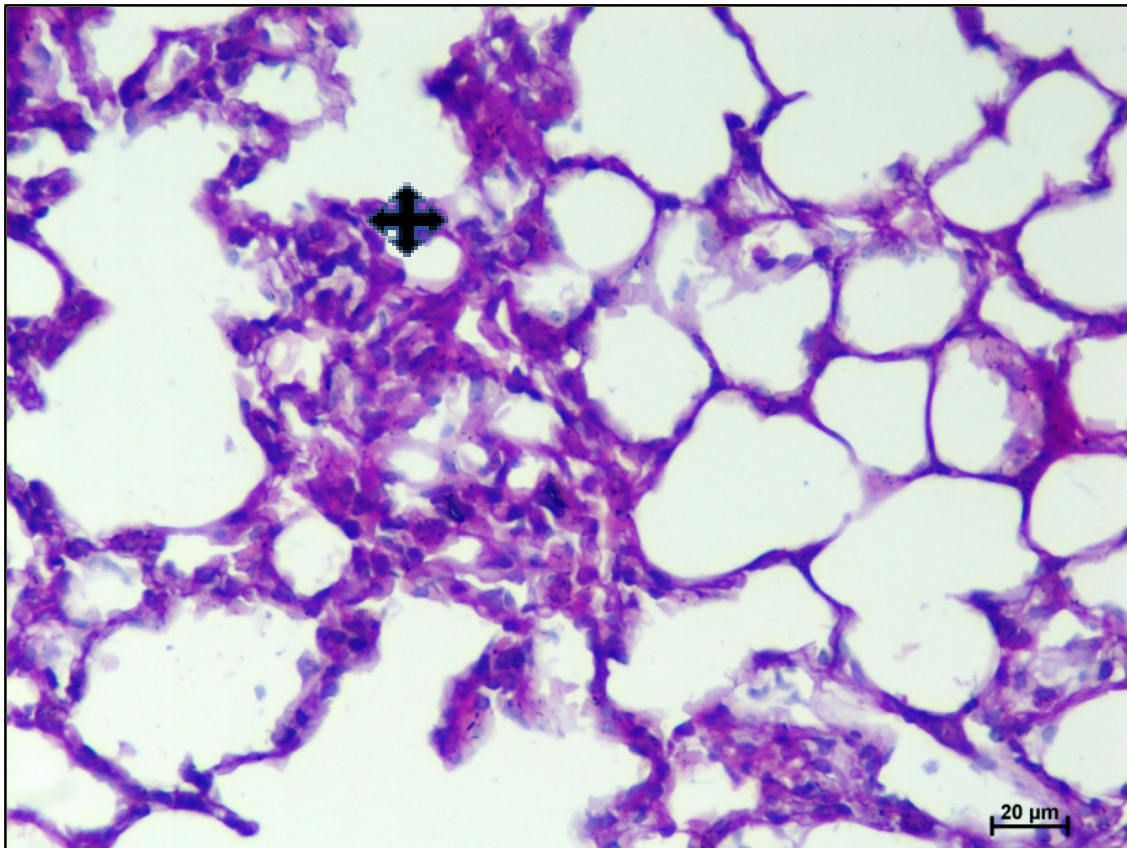

C3<sup>-/-</sup>

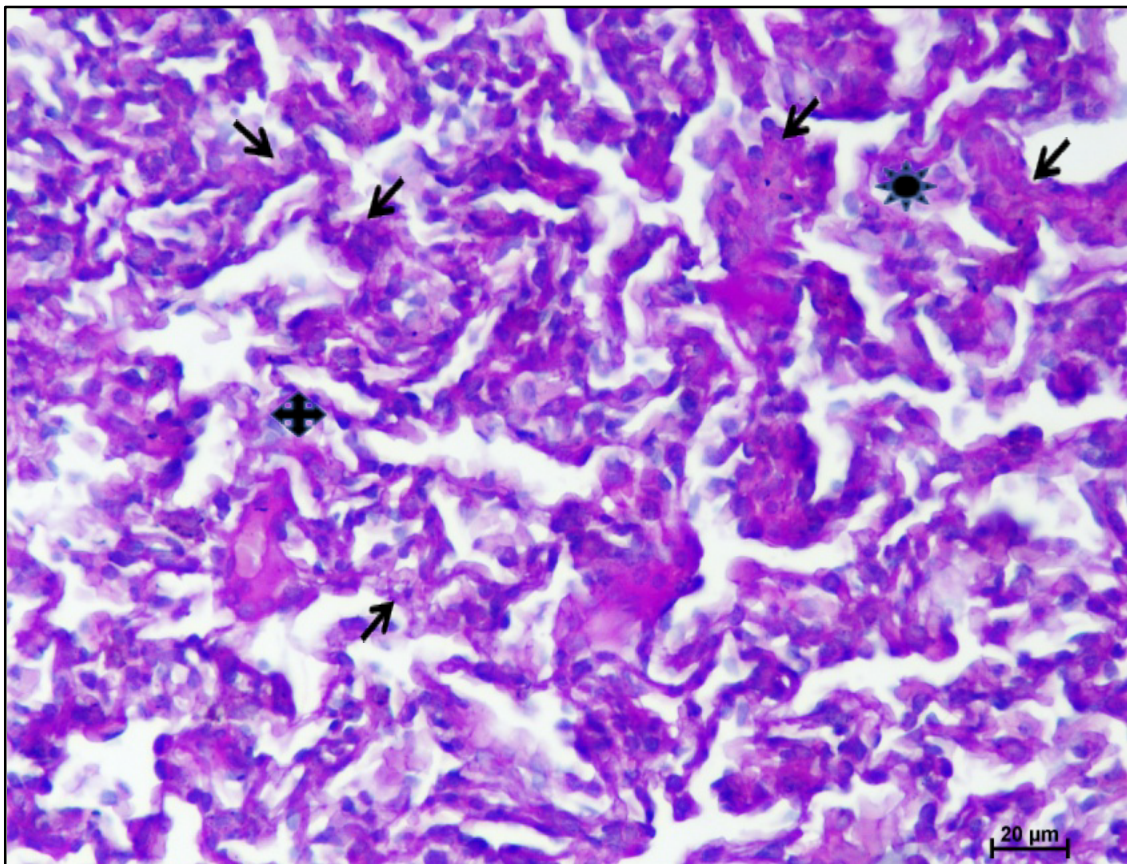

Supplement: Supplementary Figure 4 — Periodic acid-Schiff (PAS) staining of the sections of lung, spleen, liver, and kidney tissues of mice challenged with A. fumigatus at day 7 post-infection. WT (upper panel) and C3-/- (lower panel) mice were infected intravenously with 1 X 105 dose of A. fumigatus conidia, sacrificed at day 7 post-infection. The lungs, spleen, liver and kidneys were harvested for histopathology. Representative image of PAS-staining for each group (n = 4 to 5, in each group) for (A) lung, (B) spleen, (C) kidney, and (D) liver. The sections showed mass of hyphae (), hyphae (), area of very high density hyphae () and branched hyphae (). [file DataSheet_4.pdf]

Supplemental Figure 4

b : Spleen day 7 post -infection

WT

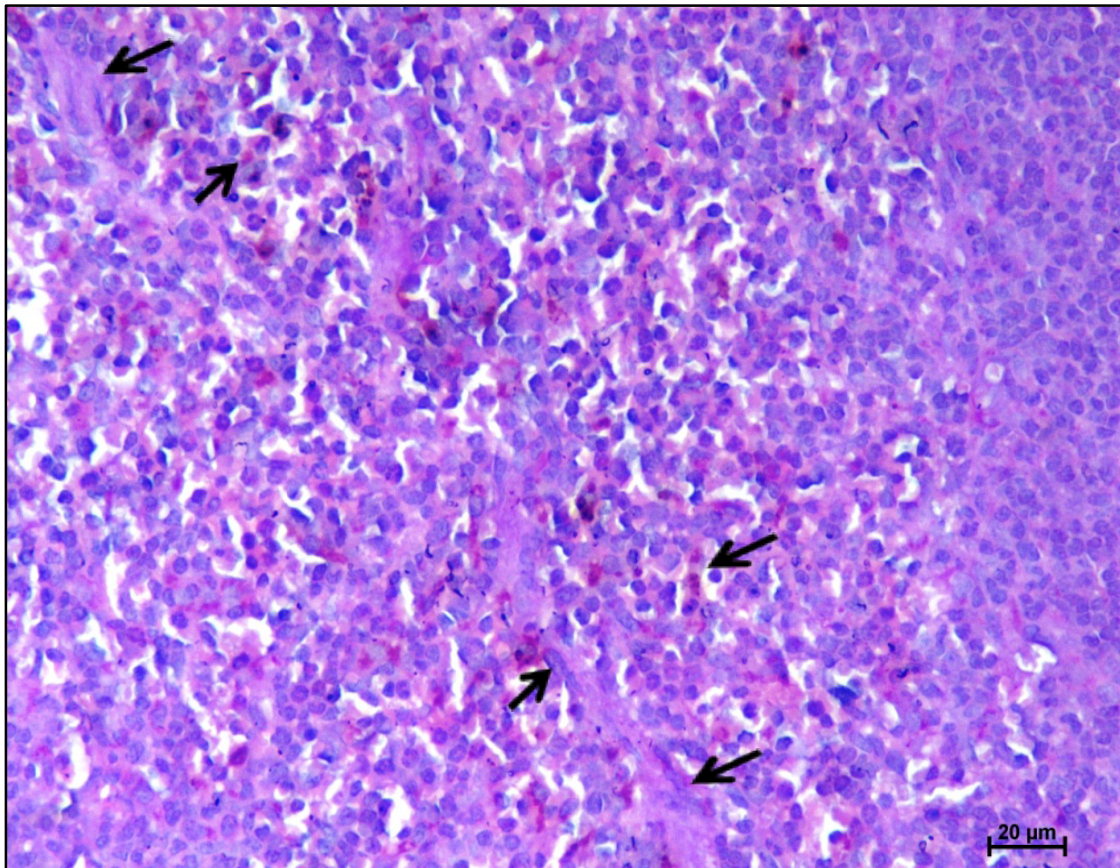

C3<sup>-/-</sup>

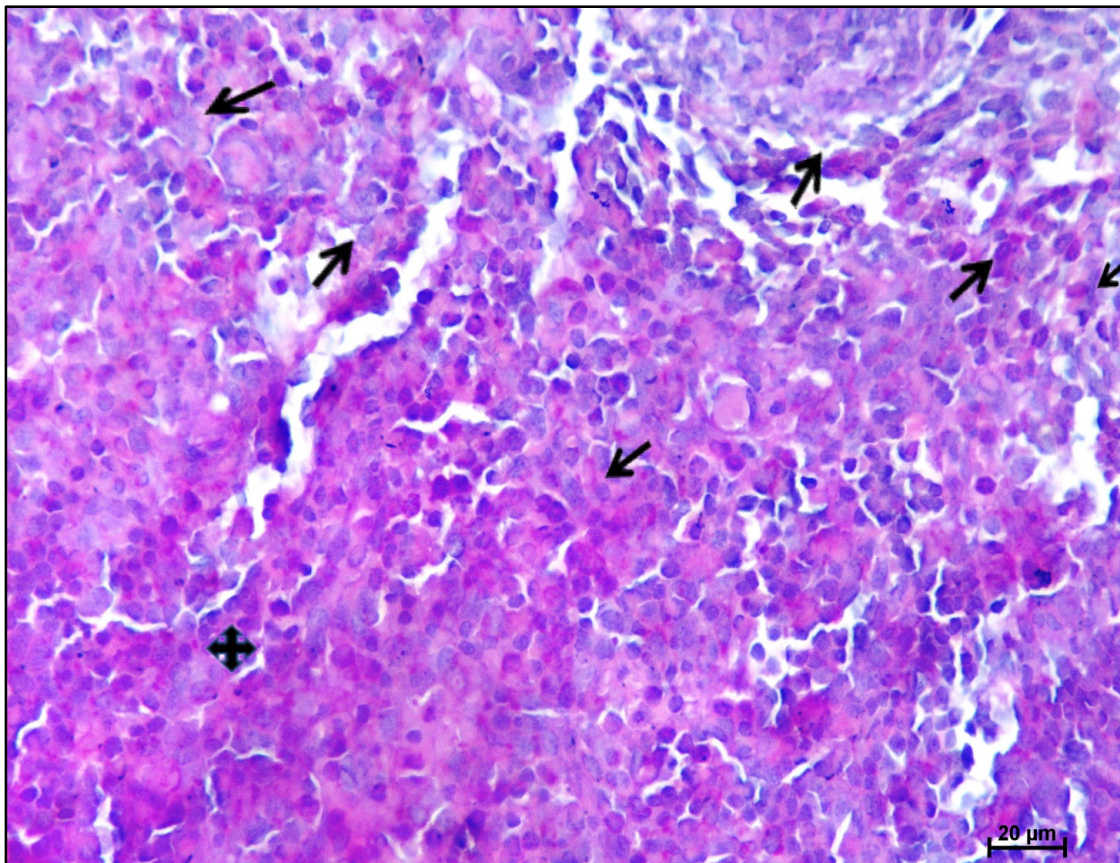

Supplement: Supplementary file 5 [file DataSheet_5.pdf]

## Supplemental Figure 4

C : Kidney day 7 post -infection

WT

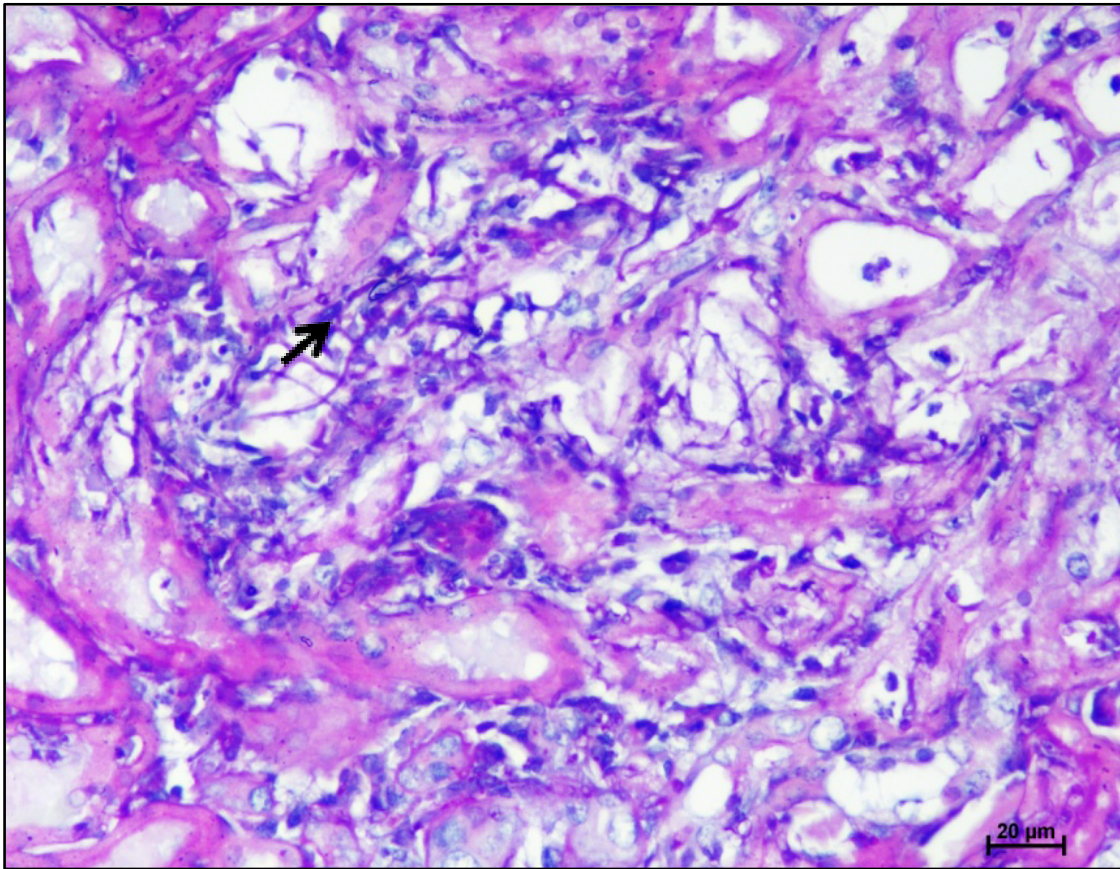

C3<sup>-/-</sup>

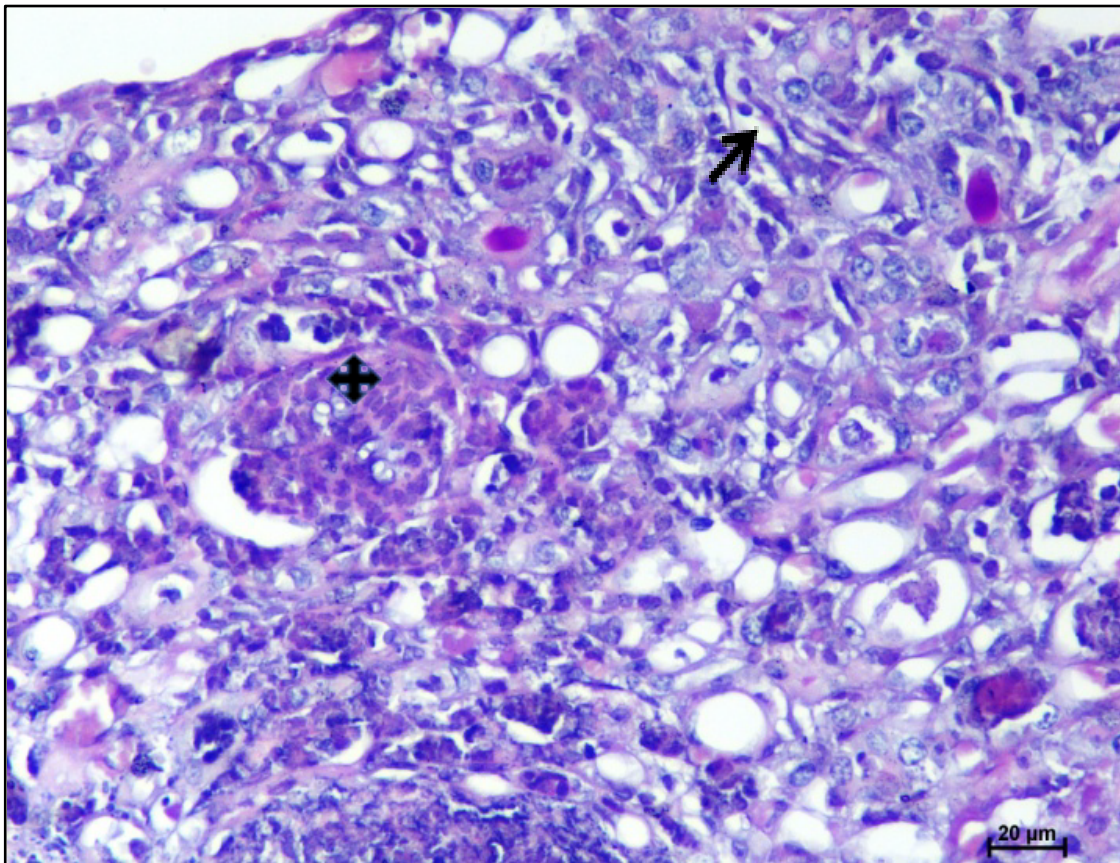

Supplement: Supplementary file 6 [file DataSheet_6.pdf]

## Supplemental Figure 4

d : Liver day 7 post -infection

WT

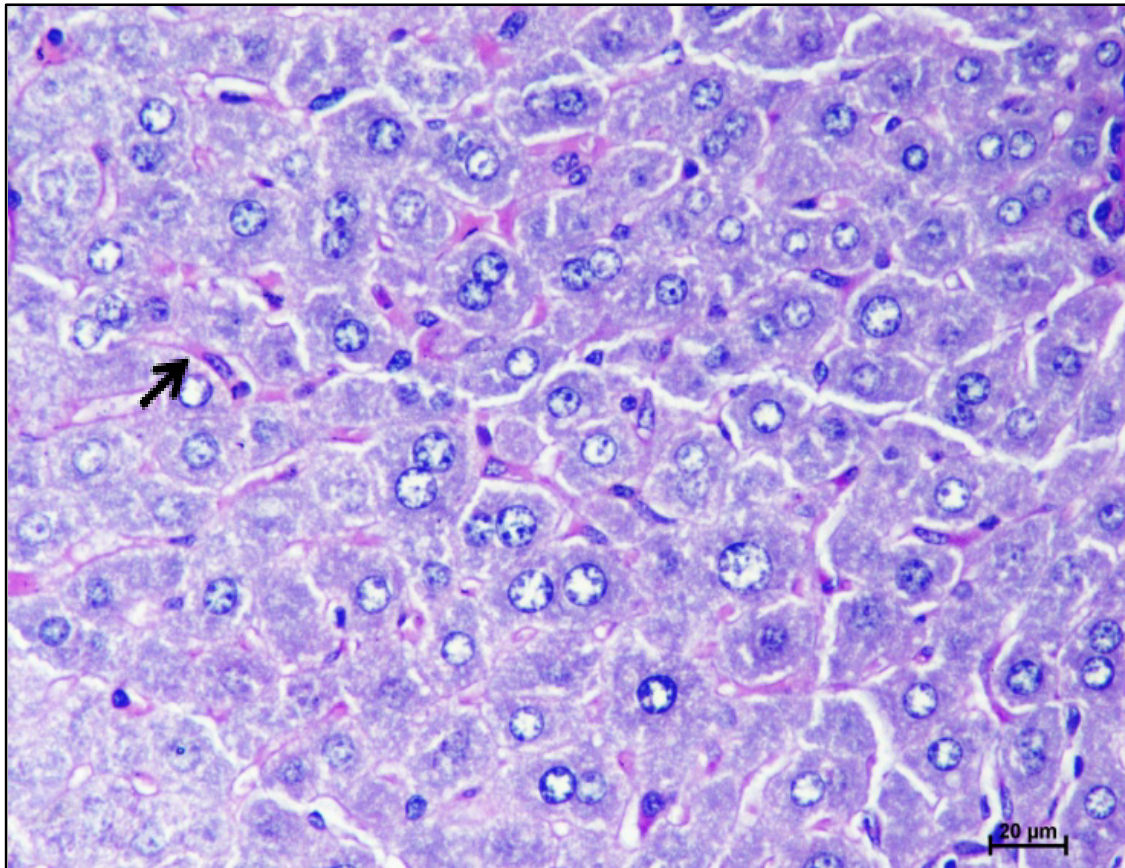

C3<sup>-/-</sup>

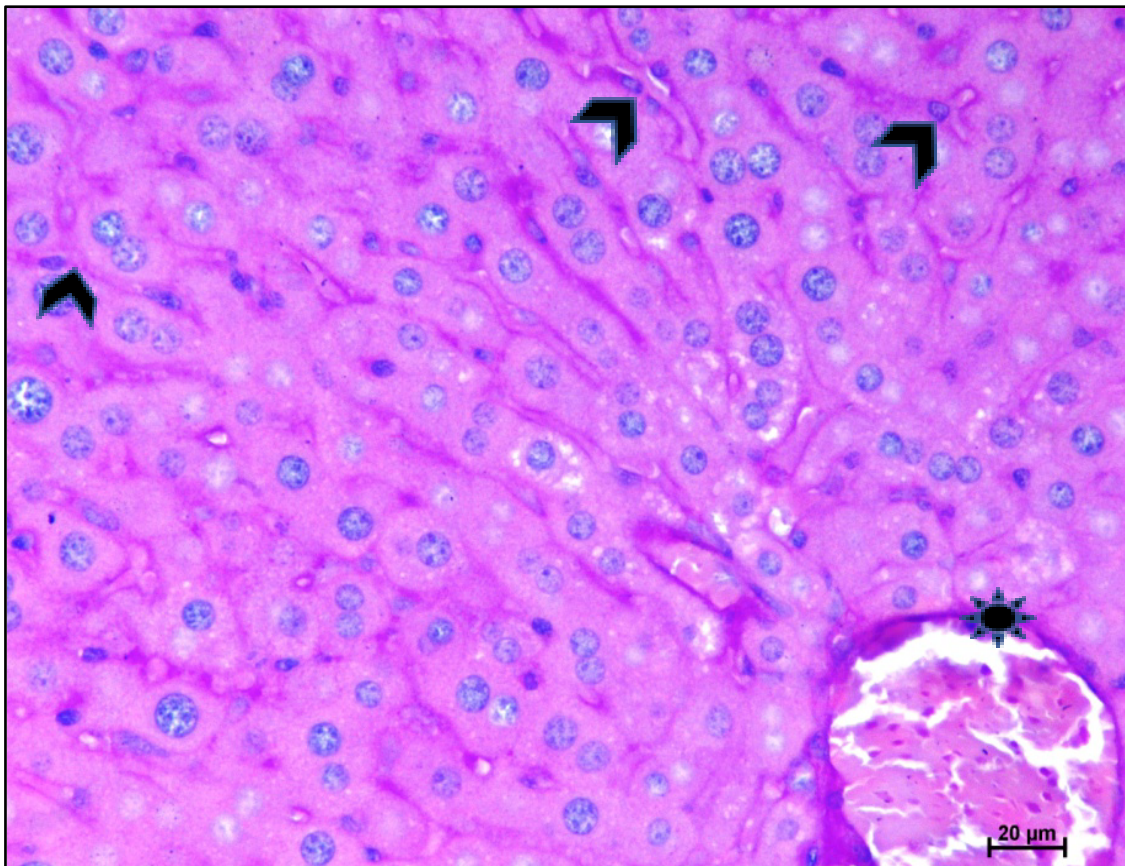

Supplement: Supplementary file 7 [file DataSheet_7.pdf]
